# Infection metallomics of pulmonary mucormycosis: multi-omics study of siderophore secretion, neutrophil recruitment, and lipid remodeling in rat lungs

**DOI:** 10.1007/s00216-026-06658-0

**Published:** 2026-07-20

**Authors:** Dominika Luptáková, Tereza Hřivnová Juříková, Miloš Petřík, Kateřina Dvořáková Bendová, Barbora Neužilová, Helena Marešová, Andrea Palyzová, Oldřich Benada, Michaela Lackner, Petr Hubáček, Vladimír Havlíček

**Affiliations:** 1 Institute of Microbiology of the Czech Academy of Sciences, Vídeňská 1083, , 142 00 Prague, Czech Republic; 2 Institute of Molecular and Translational Medicine, Faculty of Medicine and Dentistry, Palacký University, Hněvotínská 5, 779 00 Olomouc, Czech Republic; 3 Czech Advanced Technology and Research Institute, Palacký University, Šlechtitelů 241/27, 779 00 Olomouc, Czech Republic; 4 Laboratory of Experimental Medicine, University Hospital, Hněvotínská 5, 779 00 Olomouc, Czech Republic; 5 Institute for Hygiene and Medical Microbiology, Medical University of Innsbruck, 6020 Innsbruck, Austria; 6 Department of the Medical Microbiology, 2nd Faculty of Medicine, Charles University and Motol University Hospital, V Úvalu 84, 150 06 Prague, Czech Republic

**Keywords:** Mass spectrometry imaging, Mucormycosis, *Rhizopus microsporus*, Siderophores, Rhizoferrin, Infection metallomics

## Abstract

Invasive pulmonary mucormycosis caused by *Rhizopus microsporus* is often fatal, yet analytical tools to monitor fungal burden and host responses in vivo remain limited. We developed a multimodal infection metallomics workflow combining ⁶⁸Ga-desferrioxamine B PET/CT, high-resolution MALDI mass spectrometry imaging (MALDI-MSI), and targeted LC-MS to map fungal siderophore production and host tissue remodeling in a neutropenic rat model and in human samples. ⁶⁸Ga-desferrioxamine B was rapidly taken up by *R. microsporus *in vitro and accumulated in infected lungs in vivo. MALDI-MSI visualized rhizoferrin (m/z 435.1259, [M−H]⁻) and homorhizoferrin (m/z 449.1420, [M−H]⁻) confined to hyphal foci and absent from control lungs, whereas heme b was depleted and spatially segregated. Neutrophil α-defensins (RatNP-2/3/4) increased 14–60-fold and formed halos around lesions, showing an inverse trend with siderophore abundance. Lipid MSI revealed remodeling of anionic surfactant lipids, with depletion of short-chain phosphatidylglycerols and phosphatidic acid–derived species and accumulation of long-chain polyunsaturated phosphatidylglycerols and phosphatidylinositols in infected regions. Targeted LC-MS of serial urine showed that rhizoferrin and homorhizoferrin emerged by day 2, peaked on day 4 (37.2 and 15.0 µg/mL), and declined with immune reconstitution; rhizoferrin was also detected in bronchoalveolar lavage from a patient with mucormycosis. Analytically, the workflow links radiotracer uptake, high-mass-accuracy spatial ion mapping of peptides with lipids, and matrix-matched urinary LC-MS quantitation within the same infection model. This integrated platform enables spatially resolved mechanistic readouts and supports non-invasive metallophore-based diagnostics of invasive mucormycosis.

**Graphical abstract:**

**Figure:**
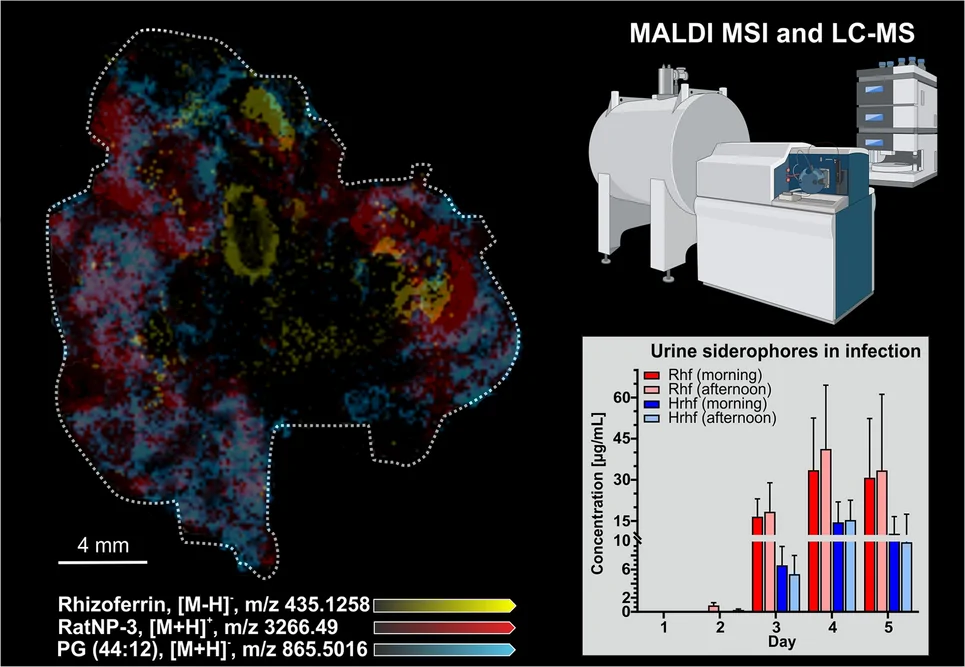

**Supplementary Information:**

The online version contains supplementary material available at https://doi.org/10.1007/s00216-026-06658-0.

## Introduction

Invasive mucormycosis, caused by molds of the order Mucorales, is an angioinvasive, rapidly progressive infectious disease characterized by extensive tissue necrosis and vascular thrombosis, with mortality approaching 100% in untreated patients and still around 25% in treated cohorts, as shown in a large therapeutic-outcome meta-analysis [1]. A mean annual incidence of 211,000 cases with 59,000 attributable deaths, together with the global distribution of mucormycosis and outbreaks after natural disasters or subsequent extreme weather events [2], its diagnostic gaps, and limited treatment options, has led to the designation of Mucorales as a high-priority group on the 2022 WHO Fungal Priority Pathogens List [3]. In Europe and other high-income settings, mucormycosis remains relatively rare and is predominantly associated with hematological malignancies, hematopoietic stem cell or solid-organ transplantation, and intensive immunosuppression [4]. In India, by contrast, the incidence is estimated to be ~ 70-fold higher than global averages, driven mainly by poorly controlled diabetes and diabetic ketoacidosis, with rhino-orbito-cerebral disease forms predominating [5], that was shown to have epidemic potential in COVID-19 survivors [6].

High-affinity iron uptake (the Fre/Fet3/Ftr1 triad) and siderophore-based iron scavenging are central, conserved virulence determinants across Mucorales, whereas secreted proteases such as an extracellular aspartic proteinase (Rmap) and alkaline protease enzyme (Arp) function as important—but likely secondary—contributors that facilitate invasion and coagulopathy in the iron-replete niches of susceptible hosts [7]. Mucorales secrete the polycarboxylate metallophore rhizoferrin (Rhf), which was first isolated from *Rhizopus microsporus* and provides a tailored system for scavenging ferric iron in otherwise iron-poor niches [8]. Recent mechanistic review on iron assimilation in Mucorales emphasizes that such metallophore/siderophore systems act in concert with high-affinity permeases and ferrioxamine receptors to overcome host nutritional immunity, tightly linking metallophore biology in *Rhizopus* spp. to mucormycosis pathogenesis and even to emerging antifungal resistance [9].

Once a proliferating mucormycete has breached the skin or mucosal barriers, innate immune cells such as macrophages and neutrophils constitute the first line of defense against the invading pathogen [10]. Among their diverse effector mechanisms, neutrophils kill microorganisms via extracellular degranulation of antimicrobial peptides and the formation of neutrophil extracellular traps. Among antimicrobial peptides, cationic defensins of 2–5 kDa, which are pre-stored in specialized cytoplasmic granules, adopt a β-sheet-rich fold and contain up to six disulfide-linked cysteine residues [11]. Defensins target negatively charged pathogen membranes, where they accumulate, preassemble, and disrupt barrier function through hydrophobic interactions between their amphipathic domains and membrane phospholipids, ultimately leading to defensin-mediated pore formation, membrane permeabilization, and cell death [11]. In rats, four neutrophil α-defensins (RatNP1–4) have been described [12].

Lung surfactant, secreted by alveolar type II epithelial cells in the form of lamellar bodies and lining the inner surface of the lung, constitutes a front-line defense during respiratory infection [13]. It is a complex lipoprotein system composed of approximately 90% lipids and 10% surfactant-associated proteins [14]. After phosphatidylcholine species (PCs), phosphatidylglycerols (PGs) and phosphatidylinositols (PIs) represent the next most abundant lipid classes [13, 14], with phosphatidic acids (PAs) serving as their integral precursors. The literature on interactions between fungal pathogens and pulmonary surfactant lipids is scarce. It has been demonstrated that PGs and PIs significantly attenuated tumor necrosis factor-α and nitric oxide production in alveolar macrophages stimulated with lipopolysaccharide (LPS) from Gram-positive bacteria [15]. Moreover, the reduction in inflammatory responses was shown to be both acyl-chain-length-specific and head-group-dependent: PGs with shorter fatty acid chains were more potent antagonists of LPS-induced cytokine production than PGs containing 16- and 18-carbon fatty acids. Abnormalities in pulmonary surfactant have been associated with various lung diseases [16], and alterations in its composition have been proposed as markers of severe respiratory failure [17] or COVID-19 [18].

Standard clinical approaches for the diagnosis of mucormycosis include microscopy with preferred staining with fluorescent brighteners calcofluor white or with histopathological staining using Grocott’s methenamine silver and periodic acid–Schiff stains, immunoassays, X-ray imaging, high-resolution computed tomography, and magnetic resonance imaging [19, 20], all of which exhibit limitations in sensitivity and specificity to varying degrees. Fungal DNA detection in serum [21], bronchoalveolar lavage (BAL) fluid [22], or tissue providing high sensitivity and specificity [23], combined with non-invasive detection of fungal siderophores in urine, is increasingly regarded as an emerging gamechanger in the early diagnosis of mucormycosis [22].

Herein, using an animal model of pulmonary mucormycosis caused by *R. microsporus*, we characterize the fungal-host interplay mediated by *Rhizopus* siderophores and rat defensins and lipids. Fungal Rhf and the newly described homorhizoferrin (Hrhf) are extracellular *Rhizopus* virulence factors secreted during fungal proliferation actively responding to host immune activation. In addition, we demonstrate how rat neutrophils, visualized via defensins, encapsulate fungal foci to slow or eliminate the proliferating pathogen that is associated with substantial remodeling of host lung tissue.

## Materials and methods

### Chemicals

Chloroform, ethanol, methanol, trifluoroacetic acid (TFA), water (HPLC grade), acetonitrile, xylene, and cacodylate buffer were purchased from VWR (Stříbrná Skalice, Czech Republic). 1,8-Bis(tetramethylguanidino)naphthalene (TMGN), 3,4-diaminobenzophenone (DABP), dihydroxybenzoic acid (DHB), hematoxylin, a silver stain (modified GMS) kit, DPX mounting medium, and microscopy glutaraldehyde solution 25% were obtained from Merck KGaA (Darmstadt, Germany). Eosin Y (in 0.5% aqueous ethanol) was purchased from Bamed s.r.o. (Litvínovice, Czech Republic). Rhf and desferrioxamine B (DfoB) standards were purchased from EMC Microcollection (Tübingen, Germany). Mouse Hepcidin 25 was purchased from PeptaNova GmbH (Sandhausen, Germany). Isoflurane was obtained from Abbott Laboratories (Abbott Park, IL, USA).

### Animal mucormycosis infection model

Experiments were conducted using 3-month-old female LEWIS rats (Envigo, Horst, The Netherlands) in agreement with our previous protocol [24]. Animals were acclimatized to laboratory conditions for 1 week prior to experimental use and housed under standard conditions on sawdust in individually ventilated cages with free access to chow and water. General health status and body weight were monitored throughout the experiments. Three to five animals per group were used in all in vivo studies. Inoculum application, radiotracer injection, and imaging were carried out under 2% isoflurane anesthesia. Rats immunosuppressed with cyclophosphamide and treated with antibiotics to prevent bacterial super-infection as previously described [24] were intratracheally infected with *R. microsporus* (∼10^7 spores in 100 μL of phosphate-buffered saline) using a TELE PACK VET X LED system equipped with a rigid endoscope (Karl Storz GmbH & Co. KG, Tuttlingen, Germany). One day before infection and throughout the infection period, urine was collected twice daily for mass spectrometry (MS) analysis. At the end of the experiment, animals were sacrificed, and lungs were immediately dissected and snap-frozen in liquid nitrogen. All samples were stored at − 80 °C until further use.

### Radio labelling, in vitro uptake of ^68^ Ga-siderophore in *R. microsporus *culture, and animal in vivo imaging

The DfoB siderophore was labeled with ^68^ Ga with radiochemical purity of 95% as previously described [25]. For kinetic in vitro uptake studies, ^68^ Ga-DfoB was incubated in triplicate with iron-deficient *R. microsporus* cultures for 5, 20, 45, and 90 min at 37 °C in Eppendorf tubes. Incubations were stopped by centrifugation for 5 min at 21,000 g, removal of the supernatant, and rapid rinsing of the fungal pellet with ice-cold Tris buffer. This washing procedure was repeated twice. After the final centrifugation and supernatant removal, Eppendorf tubes containing the fungal pellet were weighed and counted in an automatic γ-counter 2480 Wizard2 (PerkinElmer, Waltham, MA, USA). Results were expressed as percentage of applied dose per gram of fungal culture (%AD/g). For in vivo uptake specificity testing, ^68^ Ga-DfoB or ^68^ Ga-triacetylfusarinine C were incubated in triplicate with *R. microsporus* for 45 min at 37 °C in the presence and/or absence of an excess of ferri-DfoB.

Imaging of animals infected with *R. microsporus* was performed as follows. Three days post-inoculation, infected rats underwent microPET/CT imaging using an Albira PET/SPECT/CT system (Bruker Biospin Corporation, Woodbridge, CT, USA). Anesthetized rats were injected retro-orbitally with a radiolabeled tracer (^68^ Ga-DfoB) at a dose of 5–10 MBq, corresponding to 4–5 μg DfoB per animal, and positioned prone in the scanner. Static PET/CT images were acquired over 40 min starting 45 min after ^68^ Ga-DfoB injection (p.i.). A 10-min PET scan (axial field of view 148 mm) was followed by a triple CT scan (axial field of view 65 mm, 45 kVp, 400 μA, 400 projections). Scans were reconstructed with Albira software (Bruker Biospin Corporation) using maximum-likelihood expectation maximization and filtered backprojection algorithms. Reconstructed data were viewed and analyzed with PMOD software (PMOD Technologies Ltd., Zurich, Switzerland). Three-dimensional volume-rendered images were generated using VolView software (Kitware, Clifton Park, NY, USA).

### Cryo-sectioning of lung tissues

Frozen control (*n* = 3) and infected (*n* = 5) lung tissues were cryo-sectioned using a Leica CM1950 cryostat (Leica Microsystems, Wetzlar, Germany). The fresh frozen lung tissue was mounted on the specimen chuck using droplets of deionized water. After conditioning in the cryo-chamber at − 21 °C for 1 h and trimming, 20-μm-thick lung sections at coronal plane were cut and thaw-mounted onto pre-cooled conductive indium tin oxide–coated glass slides (Bruker Daltonics, Bremen, Germany) and standard microscope slides for mass spectrometry imaging (MSI) and histological evaluation, respectively. Prepared slides were stored at − 80 °C until further use. On the day of analysis, tissue sections were vacuum-desiccated for 40 min and scanned using a flatbed scanner (Epson Perfection V600, Nagano, Japan) prior to MSI and histological processing.

### Matrix-assisted laser desorption/ionization mass spectrometry imaging (MALDI-MSI) sample preparation

#### Peptide imaging

For MALDI-MSI analysis of peptides, mouse Hepcidin 25 (10 μg/mL in 40% aqueous ethanol with 1% TFA) was used as an internal standard (IS) to normalize MSI data. Mouse Hepcidin 25 was selected for its shared physicochemical characteristics with antimicrobial peptides, including a comparable molecular mass and the presence of four disulfide bonds. Additionally, mouse Hepcidin-25 generated a stable and reproducible normalization signal, showed no interference with endogenous rat tissue ions or the analytes of interest, and was not endogenously present in rat lung tissue. The IS solution was sprayed onto lung tissue sections using a SunCollect sprayer (SunChrom GmbH, Friedrichsdorf, Germany) with a Z-position of 30 mm and an offset of 5 mm. Sprayer head speed of 700 and 900 mm/min were used in the *X* and *Y* directions, respectively, applying 10 IS layers with a 2-mm line distance. Spraying flow rates of 20, 35, and 50 μL/min were used for the initial three layers and 70 μL/min for the remaining 7 layers at 2.1 bar N₂ pressure. After internal standard application, lipids were removed from lung tissues by washing with chloroform (45 mL) for 35 s, followed by rapid drying under a stream of N₂ [26]. The DHB matrix (25 mg/mL in 40% ethanol with 1% TFA) was then uniformly applied using the same spraying parameters as for the IS.

#### Carboxylate siderophore and lipid imaging

For MALDI-MSI analysis of carboxylate siderophores and lipids, a previously published sample preparation protocol was used [27]. MALDI matrices DABP and TMGN were mixed in a 3:1 ratio and dissolved in 70% acetonitrile to final concentrations of 3 and 1 mg/mL, respectively. The matrix mixture was homogeneously sprayed over tissue sections using a M3+ sprayer (HTX Technologies, USA) with the following parameters: 40 °C nozzle temperature, flow rate 90 μL/min, N₂ pressure 10 psi, spraying velocity 1100 mm/min, 10 passes with a 2 mm track spacing in a cross pattern, and a 2 s drying time between passes.

### MALDI-MSI analysis and data processing

All MSI experiments were performed on a 12 T solariX XR-2ω MALDI Fourier transform ion cyclotron resonance (FTICR) mass spectrometer (Bruker Daltonics, Billerica, MA, USA) equipped with a Smartbeam II 2 kHz laser and calibrated against red phosphorus clusters. Peptide data were acquired in positive ion mode using quadrature phase detection. The ion signal at *m*/*z* 2755.026519, corresponding to the M+2 isotope of the protonated mouse Hepcidin 25 molecule, was used for internal calibration throughout data acquisition. Pixel spectra were collected over the *m*/*z* 300–4000 range. The time-domain sample size was set to 1 Mword, providing an estimated resolving power of 390,000 at *m*/*z* 400. Optimized ion optics parameters included deflector plate voltage (220 V), funnel voltage (150 V), radiofrequency amplitude (200 Vpp), collision cell voltage (− 2.5 V), DC bias (0.8 V), collision RF frequency (2 MHz) with 1200 Vpp amplitude, time-of-flight delay (1.3 ms), quadrupole frequency (2 MHz), and Q1 mass (*m*/*z* 300).

Siderophore and lipid data were acquired in negative ion mode over the m/z 150–1500 range with optimized parameters as follows: time-domain file size 2 Mword, resolving power at m/z 400 of 190,000, deflector plate (− 220 V), plate offset (−100 V), funnel voltages (− 150 V), and RF amplitude (90 Vpp), collision cell voltage (2.5 V), DC bias (− 1.5 V), time-of-flight delay (0.7 ms), quadrupole frequency (4 MHz) and Q1 mass (m/z 200). In both methods, the laser operated at 1000 Hz (100 laser shots per position), with laser power optimized at the beginning of the study and kept constant throughout. MSI data were acquired using a 120-μm or 20-μm raster step for standard or high-spatial-resolution imaging, respectively. Spatial navigation for imaging data acquisition was set in the flexImaging v.5.0 software (Bruker Daltonics, Germany).

All MALDI-MSI data were processed in SCiLS Lab v.2023c Pro (Bruker Daltonics). Using T-Rex^2^ algorithm applying the criteria of weak spatial noise filtering, relative intensity threshold of 0.5%, and mass windows of 35 mDa for peptides and 3 mDa for siderophores and lipids, data were searched for *m*/*z* feature list. Peak lists were manually curated, and *m*/*z* values displaying the tissue distribution were further used for annotation. Peptides, siderophores, and lipids were annotated using database searches (www.uniprot.org, www.lipidmaps.org, https://ms.biomed.cas.cz/cyclobranch/docs/html/) based on mass accuracy better than 3 ppm and isotopic pattern, and normalized to mouse Hepcidin 25, and root-mean-square, respectively. Regions of interest (ROIs) were drawn manually based on the optical image. ROIs were delineated to exclude artifacts, e.g., cracks, tears, and holes, resulting from the cryo-sectioning of highly fragile and frequently damaged lung tissue, and include only intact tissue areas. In addition, major blood vessels were excluded from the ROIs to avoid their disproportionate contribution to the heme b signal and consequent bias in the analysis. Normalized average peak areas within ROIs were used for data evaluation. The normalized peak area of each compound of interest used for statistical evaluation represented the sum of its relevant ion adducts as follows: [M−H]^−^, [M−H₂O−H]^−^, [M+Na−2H]^−^, and [M+K−2H]^−^ for Rhf; [M−H]^−^ and [M−H₂O−H]^−^ for Hrhf; [M−H]^−^ for lipids; [M−H]^−^, [M+Na−2H]^−^, and [M+K−2H]^−^ for heme b; and [M+H]^+^, [M+Na]^+^, and [M+K]^+^ for RatNPs.

### Histological evaluation

In MSI-processed tissue sections, the DHB matrix was removed prior to staining by washing with absolute ethanol for 1 min. Cell nuclei were stained with hematoxylin for 1.5 min and then rinsed thoroughly with tap water for 3 min. Sections were counterstained with eosin Y for 20 s, followed by a 3-min tap water wash. Slides were then fixed in absolute ethanol for 1 min and cleared in xylene for 15 min. Visualization of *R. microsporus* was performed using silver staining according to the manufacturer’s protocol (https://tools.thermofisher.com/content/sfs/manuals/D21487~.pdf). Briefly, mucopolysaccharides of the fungal cell wall were oxidized with periodic acid solution for 5 min, followed by staining in prewarmed (62 °C) working silver methenamine solution for 22 min. Tissue sections were then toned with gold chloride solution and sodium thiosulfate solution for 30 s and 2 min, respectively, with rinses in deionized water at room temperature between steps. Sections were counterstained with eosin Y and fixed as described above, then permanently mounted with DPX medium. Tissue morphology was examined using a Leica DM2500 microscope (Leica Microsystems, Wetzlar, Germany).

### Scanning electron microscopy (SEM)

Seventy-micrometer-thick cryostat sections of the infected rat lung tissue were essentially processed using the flow fixation method, as previously described [28]. In brief, the frozen sections were transferred directly onto a frozen buffered fixative solution (3% glutaraldehyde, 0.1 M cacodylate buffer, pH 7.4) in a six-well plate at −21 °C within the cryostat chamber. Immediately after that, the plate was placed in the fridge and allowed it to warm slowly at 4 °C overnight. The floating fixed sections were then washed three times on a pre-cooled cacodylate buffer surface at 4 °C, each for 20 min. The washed sections were mounted onto wet coverslips, wrapped in fine copper mesh, and dehydrated in a graded ethanol series (25%, 50%, 75%, 90%, 96%, and 100%, 20 min each). Finally, the mounts were critical-point dried (K850, Quorum Technologies Ltd., Laughton, UK). The dried sections were mounted onto specimen stubs and sputter-coated with 3 nm of platinum. The platinum-coated samples were examined using a FEI Nova NanoSEM 450 scanning electron microscope (FEI, Brno, Czech Republic) at 5 kV, equipped with an ETD and CBS detectors. For SEM, tissue sections adjacent to those used for MSI and histopathological analyses were examined. As the analyses were performed on consecutive sections, SEM observations corresponded to the same infected tissue area but not necessarily to the exact microscopic locations analyzed by MSI or histology.

### Extraction and quantitation of siderophores in urine samples

Siderophores were extracted from rat urine samples using a modified protocol [29, 30]. Briefly, 400 μL ice-cold methanol was added to 50 μL urine, vortexed, and incubated at − 80 °C for 1 h. Samples were then centrifuged at 14,000 rpm for 10 min at 4 °C, and the supernatant was transferred to a new vial and vacuum-dried. Rhf was quantified using matrix-matched calibration curve prepared from a rhizoferrin standard compound. Hrhf was quantified using the rhizoferrin calibration curve, thus considered as semi-quantified because an authentic Hrhf standard compound was unavailable. Control urine samples (50 μL) were spiked with a standard solution of desferri-Rhf in 5% acetonitrile to yield final concentrations of 0.5, 1, 2.5, 5, 10, 25, 50, 100, 500, and 1000 ng/mL. These calibration standards were extracted using the same protocol and reconstituted in 250 μL of 5% acetonitrile prior to MS analysis.

### Liquid chromatography-mass spectrometry (LC-MS) of siderophores and data processing

Urine analyses were performed using the LC-MS-based infection metallomics approach [31]. Briefly, siderophores were separated on a Dionex UltiMate 3000 HPLC system (Thermo Scientific, MA, USA). Reconstituted urine samples were injected onto an Acquity HSS T3 column (1.8 μm, 1.0 × 150 mm; Waters, Milford, MA, USA). Analytes were eluted at a flow rate of 50 μL/min using the following gradient: 2% B for 2 min; linear increase to 70% B at 5 min and to 99% B at 8.5 min; hold at 99% B for 2.5 min; return to 2% B over 0.5 min; and re-equilibration at 2% B for 3.5 min. Solvent A was water with 0.1% formic acid, and solvent B was 99% acetonitrile with 0.1% formic acid.

MS analyses were performed on a 12 T solariX XR-2ω FTICR mass spectrometer externally calibrated against sodium trifluoroacetate clusters in electrospray ionization mode. MS data were acquired in negative ion mode over the m/z 200–1500 range. Ion optics parameters were similar to those used for MALDI-MSI of siderophores. Qualitative and quantitative data were processed using DataAnalysis v.6.0 (Bruker Daltonics). Siderophores were quantified against external matrix-matched calibration standards. Extracted ion chromatograms with a spectral width of 0.002 Da were used, and responses were calculated as integrated peak areas of the desferri-form of deprotonated ion species. LC-MS method validation parameters were adapted from the US Food and Drug Administration Guidance for Bioanalytical Method Validation (https://www.fda.gov/regulatory-information/search-fda-guidance-documents/bioanalytical-method-validation-guidance-industry) for calibration curve linearity, limit of detection (LOD), and limit of quantitation (LOQ) (Online Resource 1, Table S1). The LOD and LOQ for Rhf in urine were determined to be 8 and 26 ng/mL, respectively.

### Statistics

Statistical analyses were performed using GraphPad Prism 10.0.1 for Windows (GraphPad Software, San Diego, CA, USA). Descriptive statistics included means and standard deviations (SD), coefficient of variation, and 95% confidence intervals. Nonlinear regression using the ROUT method was applied to identify and exclude potential outliers. Data passed the Shapiro-Wilk normality test and were therefore analyzed using parametric tests. To compare control and infected lung tissue groups, unpaired two-tailed *t*-tests assuming equal variances were used. To test for differences among multiple compounds within infected and control groups, ordinary one-way ANOVA followed by the uncorrected Fisher’s LSD multiple comparison test was performed. Statistical significance was reported as exact *p*-values.

## Results

### Monitoring the extent of *R. microsporus *burden in lung tissues by multimodal imaging

**Fig. 1:**
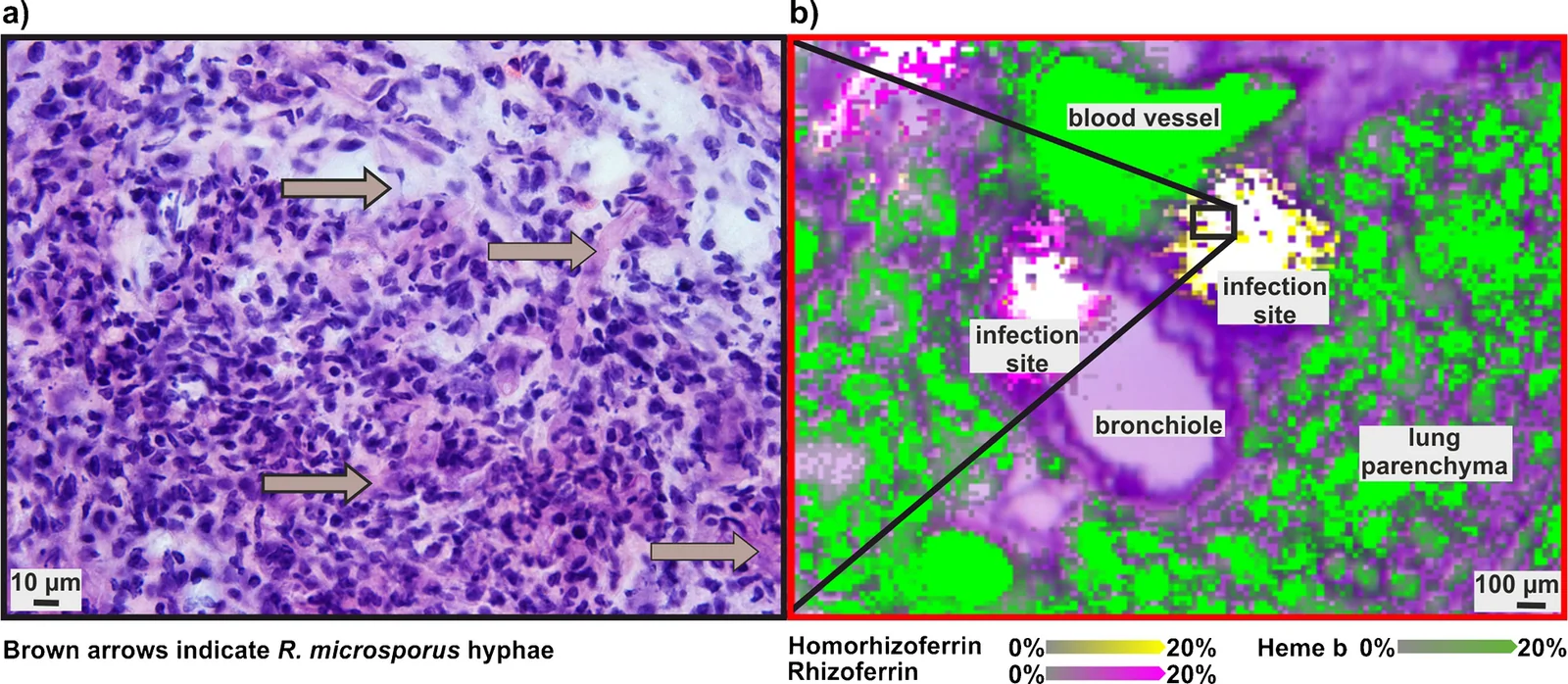
Multimodal imaging of *R. microsporus*–infected lung tissue. **a** Zoom in the optical microscopy image after hematoxylin and eosin staining showing dense fungal hyphae (brown arrow) in the lung tissue. **b** MALDI-MSI overlay with optical microscopy. High-spatial-resolution ion images showing the distribution of *R. microsporus* siderophores—rhizoferrin (pink shading) and homorhizoferrin (yellow shading)—and heme b (green shading) are co-registered with the histological image. Rhizoferrin and homorhizoferrin signal was detected in regions containing histologically identified fungal hyphae. Representative ion images were acquired at 20 µm raster step and root-mean-square normalized. Relative ion intensities are displayed using single-color scales linearly scaled from 0 to 20%

**Fig. 2:**
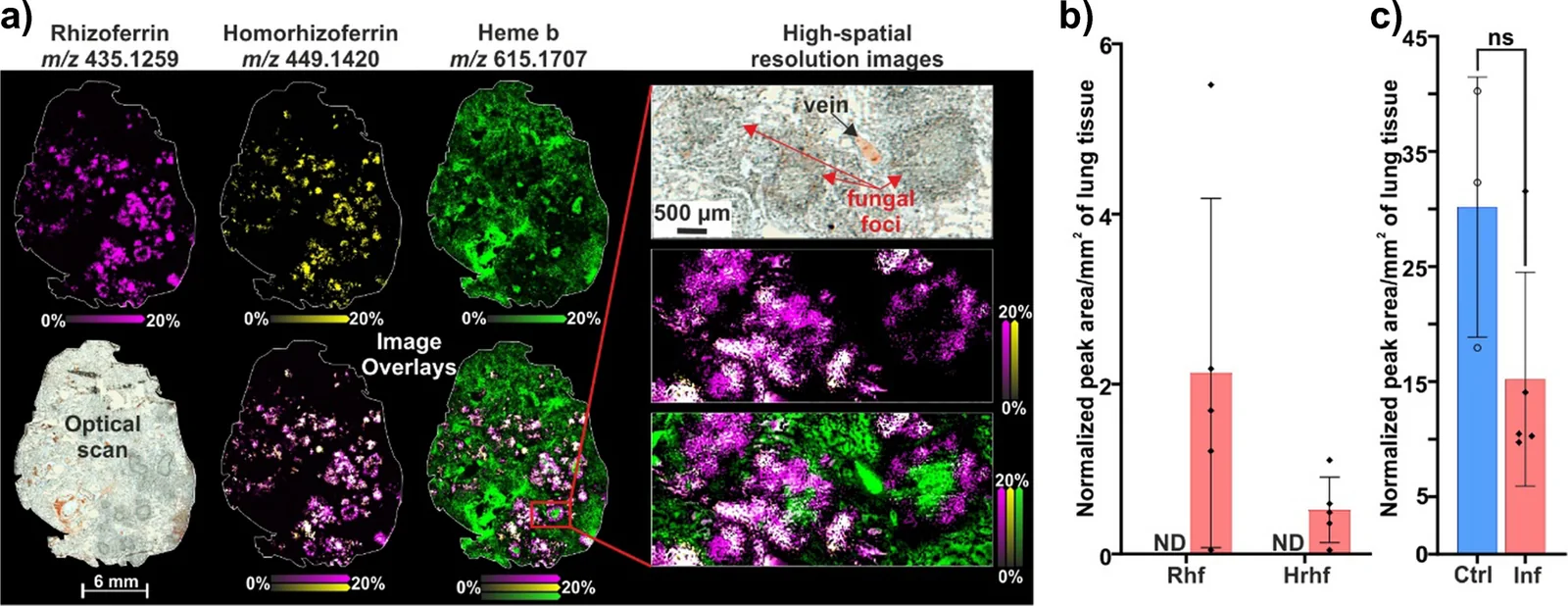
Visualization and relative quantitation of *R. microsporus* siderophores and heme b in infected and control lung tissue sections. **a** MALDI-MSI of infected lung tissue showing the relative ion intensities of rhizoferrin (Rhf, m/z 435.1259, [M−H]⁻), homorhizoferrin (Hrhf, m/z 449.1420, [M−H]⁻), and heme b (m/z 615.1707, [M−H]⁻). Representative images of the whole lung tissue section and of a discrete fungal focus were acquired at 120 µm and 20 µm raster step, respectively, and root-mean-square-normalized. Relative ion intensities across the whole tissue section are displayed using single-color scales linearly scaled from 0 to 20%. **b** Bar graph showing the relative abundance of Rhf and Hrhf in control (*n* = 3) and infected (red, *n* = 5) lung tissue sections extrapolated from delineated region of interests. Rhf and Hrhf were not detected (ND) in control lungs. **c** Bar graph showing the relative abundance of heme b (m/z 615.1707, [M−H]⁻) in control (Ctrl, blue, *n* = 3) and infected (Inf, red, *n* = 5) lung tissue sections extrapolated from delineated region of interests. Data are shown as mean ± standard deviation

The gallium-labeled xenosiderophore ⁶⁸Ga-DfoB showed rapid uptake by *R. microsporus* spores in vitro (Online Resource 1, Fig. S1a,b), and PET/CT imaging demonstrated pronounced radiotracer accumulation predominantly in the lungs, with additional signal in the excretory organs (intestines, kidneys, and urinary bladder) 45 min p.i. (Online Resource 1, Fig. S1c,d). Histopathology of infected lungs revealed extensive fungal proliferation with dense hyphal networks, and abundant spores distributed throughout the tissue (Online Resource 1, Fig. S2a–c) that was additionally confirmed by detailed imaging of hyphae in the lung tissue by SEM (Online Resource 1, Fig. S2d,e). *R. microsporus* formed sharply demarcated foci with hyphae concentrated at the periphery and central zones displaying tissue degeneration, cell death, and hemorrhage (Fig. 1a). High-spatial resolution MALDI-MSI detected Rhf (m/z 435.1259, [M−H]⁻), whose spatial distribution co-localized with fungal hyphae (Fig. 1b), and a single additional co-localized ion at m/z 449.1420 assigned to homorhizoferrin (Hrhf) (Fig. 1b, Fig. 2a). Hrhf is described here for the first time as a characteristic siderophore of *R. microsporus*. Neither Rhf nor Hrhf was detected in control lung tissue (Fig. 2b). In contrast, the relative abundance of heme b (m/z 615.1707, [M−H]⁻) was reduced in infected lungs compared with controls, and its distribution did not overlap with siderophore-rich regions but corresponded to erythrocyte-rich areas on histology (Fig. 1b, Fig. 2c).

### Host neutrophil peptides respond to *R. microsporus* lung infection

**Fig. 3:**
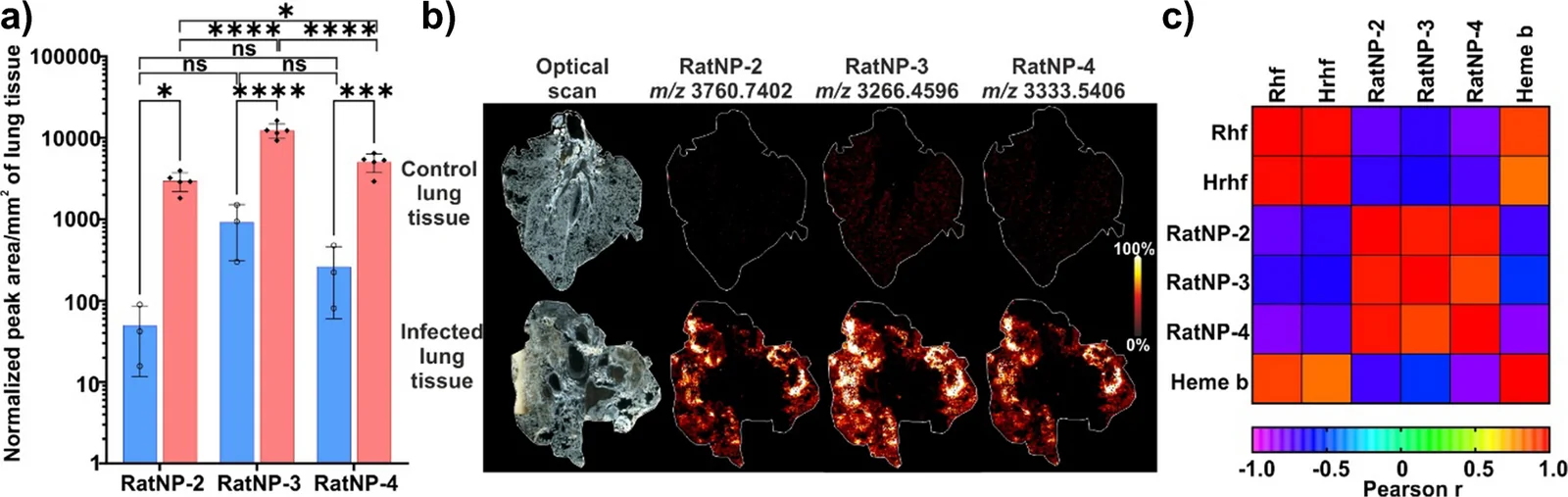
MALDI-MSI analysis of the rat immune response to *R. microsporus* lung infection and correlation of host-pathogen molecular markers. **a** Bar graph comparing the relative abundance of RatNPs between control (blue, *n* = 3) and infected (red, *n* = 5) lung tissue sections. Data are presented as mean ± standard deviation. *, *p* ≤ 0.05; ***, *p* ≤ 0.001; ****, *p* ≤ 0.0001; ns, not significant. **b** MALDI-MSI of control and infected lung tissue sections showing the relative ion intensities of RatNP-2 (m/z 3760.7402, [M+H]⁺, M+2 isotope), RatNP-3 (m/z 3266.4596, [M+H]⁺, M+2 isotope), and RatNP-4 (m/z 3333.5406, [M+H]⁺, M+2 isotope). Representative images were acquired at 120 µm raster step and normalized to the peak area of the molecular ion of mouse Hepcidin 25. Normalized ion intensities are displayed using a fire-color scale linearly scaled from 0 to 100%. **c** Correlation matrix showing Pearson correlation coefficients between RatNPs and the siderophores rhizoferrin (Rhf) and homorhizoferrin (Hrhf), as well as heme b. Results are visualized using a rainbow-color scale. Fold-change values reported in the “Results” were calculated from ROI-derived mean normalized peak areas

In this model, neutropenia was induced prior to inoculation to facilitate the development of pulmonary mucormycosis, followed by spontaneous immune reactivation approximately 3 days later. Neutrophils accumulated predominantly at the infection site, forming dense rims around regions rich in *R. microsporus* hyphae and spores (Online Resource 1, Fig. S2c). Neutrophils in hematoxylin and eosin Y-stained sections were identified on morphological grounds, mainly by their characteristic multilobed nuclei; no neutrophil-specific staining was performed, and the rat neutrophil peptides (RatNPs)-MSI signal was therefore interpreted as a complementary molecular marker of neutrophil-rich regions. The magnitude of the neutrophil response was assessed by MALDI-MSI detection of RatNPs. Of the four known RatNPs, three—RatNP-2 (m/z 3760.7402, [M+H]⁺, M+2 isotope), RatNP-3 (m/z 3266.4596, [M+H]⁺, M+2 isotope), and RatNP-4 (m/z 3333.5406, [M+H]⁺, M+2 isotope)—were detected in lung tissue in their protonated forms (Online Resource 1, Table S2), as well as in sodiated and potassiated adducts (Online Resource 1, Fig. S3). Compared with control lungs, which showed similar low basal RatNP levels, infected lungs exhibited markedly increased RatNP signals across infected tissues (Fig. 3). RatNP-3, RatNP-4, and RatNP-2 increased by 14-fold (*p* < 0.0001), 19-fold (*p* = 0.0002), and 60-fold (*p* = 0.0114), respectively, when comparing mean normalized peak areas between infected (*n* = 5) and control (*n* = 3) animals. These fold-increases were calculated from mean normalized peak areas within ROIs; control tissues showed low but measurable basal RatNP signals, whereas the ion images were scaled independently to visualize spatial distribution. RatNP-3 showed the highest overall abundance, exceeding RatNP-4 by 2.4-fold (*p* < 0.0001) and RatNP-2 by 4.2-fold (*p* < 0.0001), while RatNP-4 levels were 1.7-fold higher than RatNP-2 (*p* = 0.0310) (Fig. 3a, b; Online Resource 1, Table S3). MALDI-MSI further revealed heterogeneous RatNP distribution within infected lungs. The highest peptide levels were confined to neutrophil-rich regions surrounding infection foci, whereas more uniform, moderate RatNP signals were observed in other minimally affected areas (Online Resource 1, Fig. S4). Pearson correlation analysis (*p* ≤ 0.05) showed strong positive correlations among Rhf and Hrhf, and likewise among all three RatNPs (Fig. 3c; Online Resource 1, Table S4). In contrast, siderophore abundance and RatNP abundance displayed a strong inverse correlation trend.

### *R. microsporus*–induced lipid remodeling of the infected lung tissue

**Fig. 4:**
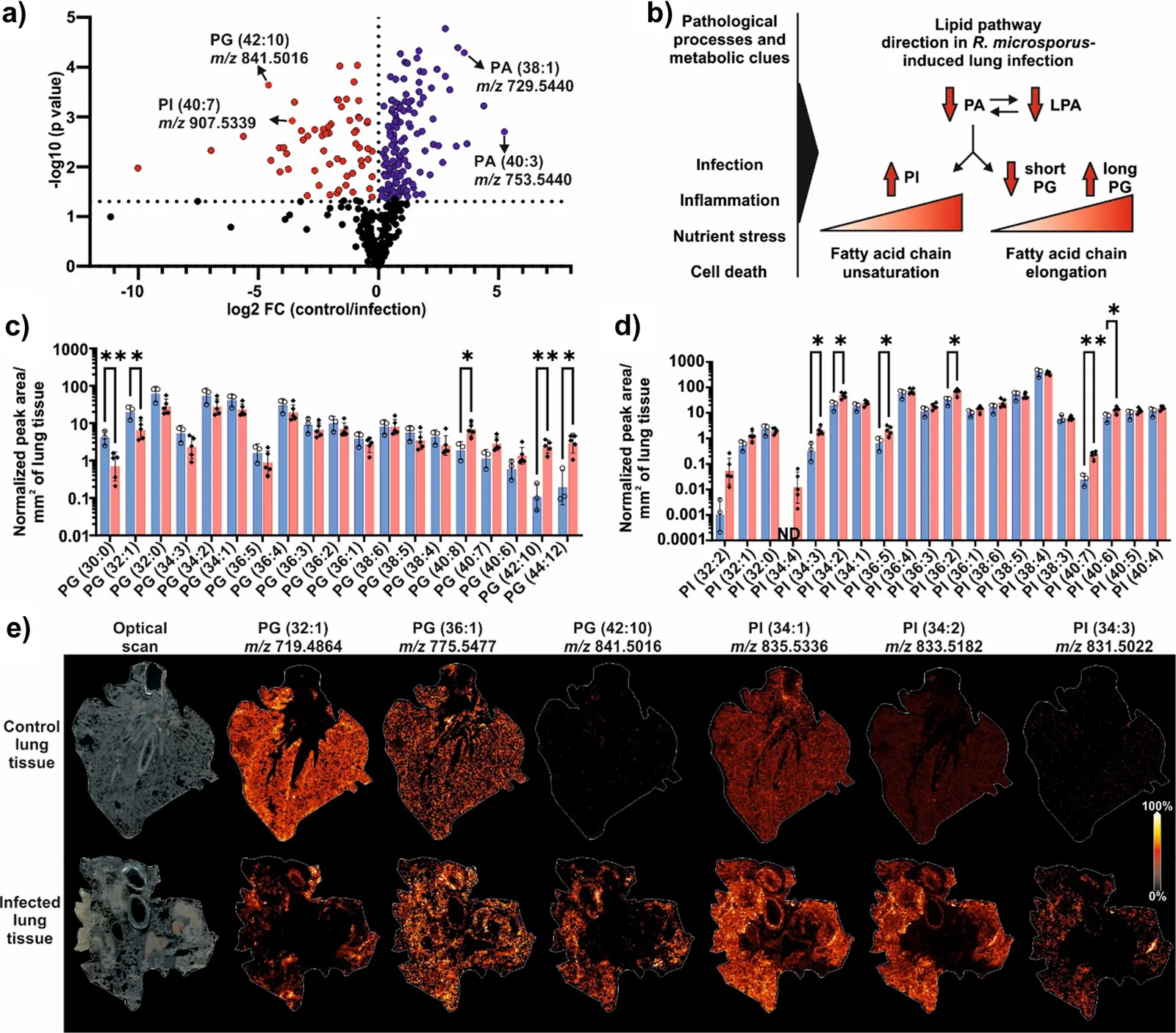
*Rhizopus microsporus* infection–induced changes in the lung lipid profile. **a** Volcano plot generated from all 418 m/z features detected by MALDI-MSI in negative ion mode over the m/z 150–1500 range, illustrating significant differences between control (*n* = 3) and infected (*n* = 5) lung tissue sections. **b** Schematic overview of characteristic alterations in lung lipid distribution associated with histologically and molecularly defined pathological processes, including infection, inflammation, nutrient stress due to *R. microsporus* iron acquisition, and cell death. **c**, **d** Bar graphs showing differences in the relative abundance of identified phosphatidylglycerols (PGs, c) and phosphatidylinositols (PIs, d) between control and infected lung tissue sections. Data are expressed as mean ± standard deviation on a logarithmic scale. *, *p* ≤ 0.05; **, *p* ≤ 0.01; ***, *p* ≤ 0.001. **e** MALDI-MSI ion images displayed as [M−H]^−^ molecular ions showing the spatial distribution of selected PG and PI species in control and infected lung tissue sections. Representative images were acquired at 120 µm raster step and root-mean-square normalized. Relative ion intensities are displayed using a fire-color scale linearly scaled from 0 to 100%

MALDI-MSI analysis in negative ion mode initially detected 2122 m/z features. After manual curation, 418 lung-relevant m/z values were retained for multiple-comparison statistical analysis (Fig. 4a). Among these, 79 lipid species were annotated by accurate mass (mass error < 2 ppm) and assigned to PGs, PIs, PAs, lysophosphatidic acids (LPAs), cyclophosphatidic acids (CPAs), and fatty acids (FAs) (Online Resource 1, Table S5). In all comparisons, control lungs were used as the reference state. In infected lungs, the most pronounced alterations were observed in PG, PI, and PA classes (Fig. 4a, b). Infection was associated with a marked redistribution of PG species according to acyl-chain length (Fig. 4c, e). PG species with shorter total acyl chains (≈30–38 carbons) were strongly decreased in infected tissue, whereas PGs with elongated, highly polyunsaturated acyl chains (> 40 carbons) became significantly enriched relative to controls. Likewise, fungal infection and inflammation induced a global increase in PI species in infected lungs (Fig. 4d). At a fixed total carbon number, infected tissue consistently favored more unsaturated PI species, particularly within the 32- and 34-carbon series (Fig. 4d, e). By contrast, all detected PA, LPA, and CPA species were markedly depleted in infected lungs. The largest decreases affected less-unsaturated species, including lipids carrying 18-carbon acyl, which remained relatively abundant in control tissue.

### Non-invasive and invasive monitoring of pulmonary mucormycosis in rat urine and human BAL fluid

**Fig. 5:**
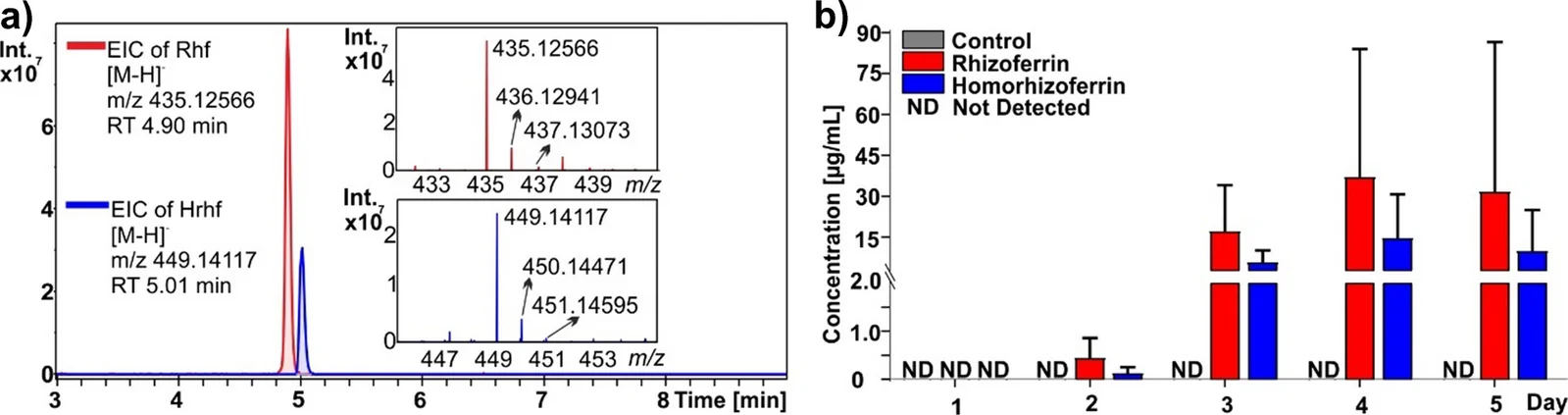
Quantitation of rhizoferrin (Rhf) and homorhizoferrin (Hrhf) in control and *R. microsporus*–infected rat urine by liquid chromatography-mass spectrometry (LC-MS). **a** Representative extracted ion chromatograms (EICs) of Rhf and Hrhf obtained by LC-MS analysis of urine from an *R. microsporus*–infected rat. **b** Quantitation of Rhf and Hrhf in urine from control (*n* = 3) and infected (*n* = 5) rats. Data are shown as mean ± standard deviation. Both siderophores were not detected (ND) in control urine samples. RT, retention time

In the present animal study, morning and afternoon urine samples were collected daily over a 5-day experimental period. Following validation of the LC-MS method (Online Resource 1, Table S1), analysis of extracted urine samples revealed the presence Rhf (m/z 435.1257, [M−H]⁻) and Hrhf (m/z 449.1412, [M−H]⁻) (Fig. 5a). Molecular identification was further confirmed by fragmentation spectra showing characteristic neutral losses of H_2_O and CO_2_ from the precursor ions (Online Resource 1, Fig. S6). Both Rhf and Hrhf were first detected in urine collected on the afternoon of day 2 (Online Resource 1, Fig. S7a), at concentrations of 0.91 µg/mL and 0.27 µg/mL, respectively. The average daily concentrations of both siderophores then increased rapidly and peaked on day 4 (Fig. 5b), reaching 37.2 µg/mL for Rhf and 15.0 µg/mL for Hrhf. Despite ongoing infection, siderophore levels declined on day 5 in four out of five monitored rats (Online Resource 1, Fig. S7b). The exception was rat 5, which showed persistently high urinary siderophore concentrations; the corresponding tissue data were consistent with the most extensive pulmonary infection and the weakest RatNP signal among the infected animals, supporting biological heterogeneity. Thus, the LC-MS assay provides a temporal analytical counterpart to MALDI-MSI by converting tissue-localized siderophore production into serial and quantitative urinary data. Using the same chromatographic method, we further demonstrated that Rhf can also be detected in a proximal fluid directly sampling the site of infection: Rhf was identified in a BAL sample from a patient with pulmonary mucormycosis (Online Resource 1, Fig. S8).

## Discussion

The rapid, focal pulmonary accumulation of ⁶⁸Ga-DfoB together with its rapid elimination via the urinary tract is consistent with active, selective uptake of ⁶⁸Ga-DfoB by *R. microsporus* and efficient renal clearance by the host, implying a short intravascular residence time of the radiotracer [25]. Building on earlier work by Drechsel and Winkelmann, who first identified Rhf and a series of related polycarboxylate siderophores from *R. microsporus* in fermenter cultures [32], our data move Hrhf from the category of a fermentation-associated analog to a bona fide in vivo virulence metabolite. High-resolution MALDI-MSI demonstrates that both Rhf and Hrhf are produced within infected lung tissue and co-localize with hyphae-rich foci, whereas neither compound is detectable in control lungs. The marked reduction of heme b signal in infected lungs, its spatial separation from Rhf/Hrhf “hotspots,” and the restriction of heme b to erythrocyte-rich regions on histology are fully consistent with the current model that Mucorales exploit heme and hemoglobin as major iron sources within necrotic, hemorrhagic lesions. Clinical and experimental studies have shown that angioinvasion grants Mucorales access to hemoglobin, which is then processed via fungal heme-uptake pathways and heme oxygenases to liberate iron [33]. Our imaging data fit this framework by suggesting local heme b consumption or degradation in hyphae-dense regions while residual heme b is confined to areas where vascular integrity and erythrocytes are still present. The prominent, focal production of Rhf and Hrhf dovetails with the concept that Mucorales have specialized toward polycarboxylate siderophores that evade lipocalin-2 (Lcn2) mediated nutritional immunity. Lcn2 binds ferric catecholate and, to a lesser extent, hydroxamate siderophores with high affinity, but does not efficiently recognize rhizoferrin-type ligands, which are therefore classified as “stealth” or siderocalin-resistant siderophores [34]. Within this context, our demonstration that *R. microsporus* avidly takes up ⁶⁸Ga-DfoB in vivo links imaging to the long-recognized clinical observation that DfoB therapy dramatically increases the risk of mucormycosis [35].

The principal analytical advance of this work is the integration of chemically selective radiotracer uptake, high-mass-accuracy MALDI-FTICR-MSI, peptide normalization using an exogenous internal standard, ROI-normalized lipid and peptide biomarkers, and matrix-matched urinary LC-MS into one infection metallomics workflow. This design allows pathogen-derived metallophores, host antimicrobial peptides, heme b, and lipid remodeling to be interpreted as connected spatial and temporal readouts.

Transient neutropenia is one of the most important clinical risk factors for mucormycosis, and reversal of neutropenia is a central pillar of management in patients with invasive disease [36]. Our experimental design—induction of neutropenia before *R. microsporus* inoculation followed by spontaneous immune reconstitution—thus closely mirrors a common clinical trajectory in hematologic and transplant patients. The strong perilesional accumulation of neutrophils we observe around hyphae- and spore-rich foci in reconstituted animals is in line with in vivo models showing that robust phagocyte recruitment to Mucorales spores is a key determinant of protection, whereas impaired recruitment or persistent neutropenia predispose to progressive disease [37]. To the best of our knowledge, our study is the first to map RatNP-2/3/4 directly in infected lung tissue and to link their abundance to a defined fungal pathogen. Biochemically, this spatially describes neutrophil degranulation at the host-fungus interface; biomedically, it suggests a way to monitor immune reconstitution within infected tissue; analytically, it establishes RatNPs as endogenous peptide markers that can be co-registered with siderophores and lipid changes in the MSI experiment. The massive fold-increases we observe up to 60-fold for RatNP-2—with RatNP-3 the most abundant overall—are consistent with strong degranulation of α-defensin-rich neutrophils at the margins of *R. microsporus* lesions. While our data do not directly demonstrate causality (e.g., that RatNPs limit siderophore production or vice versa), they show that maximal siderophore accumulation and maximal neutrophil defensin deployment rarely coincide in the same microdomains. Although RatNP-3 was the most abundant and RatNP-2 the least abundant peptide, there is currently no evidence for *R. microsporus*–specific differential regulation of individual RatNPs. The observed rank order likely reflects intrinsic differences in gene expression and peptide stability among rat α-defensins established during neutrophilic myelopoiesis, as reported for RatNP 1–4 and human HNP1–4 [12]. The biologically relevant point in this context is the concerted, strong upregulation of all three neutrophil α-defensins at sites of invasive mucormycosis.

Within the constraints of our negative ion MALDI-MSI acquisition, the PG, PI, PA, LPA, and CPA species that change during infection are, to the best of our knowledge, interpreted as host-derived surfactant and cellular phospholipids [38]; major fungal virulence lipids such as sterols and complex sphingolipids [39, 40] are therefore not resolved spatially here. Relative to uninfected lungs, *R. microsporus* infection is characterized by a coupled loss of canonical, short-chain anionic surfactant lipids (short, weakly unsaturated PG and PA/LPA/CPA species carrying C18 chains) and a reciprocal accumulation of long-chain, highly polyunsaturated PG and PI species (> C40, ≥ 8 double bonds) in infected foci. This pattern follows the established roles of anionic surfactant phospholipids in host defense. Palmitoyl-oleoyl-phosphatidylglycerol (POPG) and PI antagonize activation of multiple Toll-like receptors (TLR1/2/4/6), suppress LPS-induced cytokine release from alveolar macrophages, and directly inhibit respiratory syncytial virus and influenza A infection in vitro and in vivo [15]. Clinical and experimental studies in acute respiratory distress syndrome and severe pneumonia similarly report quantitative depletion of surfactant PG, profound alterations in the fatty acid composition of surfactant phospholipids, and impaired surface activity, indicating that selective PG loss is a hallmark of severe inflammatory lung injury [41]. Conversely, the enrichment of long, highly polyunsaturated PG and PI species is consistent with enhanced incorporation of C20–C22 polyunsaturated fatty acids into membrane phospholipids, and supporting rapid remodeling of immune-cell membranes in inflamed tissue [42]. Phospholipid metabolism is actively reprogrammed during innate immune responses, with targeted changes in acyl-chain length and unsaturation shaping membrane plasticity, receptor clustering and downstream signaling [43].

The urinary time-course of Rhf and Hrhf in our neutropenic rat model indicates that these siderophores primarily reflect metabolically active fungal burden under a dynamically changing immune status. Rhf and Hrhf first become detectable only from the afternoon of day 2 and then rise steeply to a maximum on day 4. Their subsequent decline on day 5 in most animals occurs while pulmonary infection persists histologically, but coincides with neutrophil recovery and accumulation of neutrophil-derived antimicrobial peptides in infected regions in this model. Together with our in vitro data [29] showing that siderophores, once present in the circulation, are excreted into urine within hours, this pattern supports a model in which the delay to first urinary detection is driven mainly by fungal germination and induction of siderophore biosynthesis, whereas the falling phase reflects neutrophil-mediated damage to hyphae that can no longer sustain high-level siderophore production.

As a preliminary, model-dependent interpretation, the rising phase of the in vivo curve can be used to approximate urinary siderophore kinetics. The ≈40-fold increase in urinary Rhf between ~ 48 h (0.91 µg/mL) and ~ 96 h (37.2 µg/mL) after inoculation gives an apparent doubling time on the order of 8–10 h. This back-extrapolation should be regarded as a hypothesis-generating estimate, because it is derived from a small animal cohort, assumes a relationship between urinary siderophore concentration and metabolically active fungal biomass, and has not yet been validated against denser time-course sampling or independent fungal burden measurements. Under comparable experimental conditions, urinary Rhf may therefore have potential for early non-invasive detection, but the timing, diagnostic threshold, and clinical transferability require confirmation in future studies.

## Conclusion

We established an integrated analytical framework that combines siderophore-targeted imaging, peptide and lipid MALDI-MSI, and targeted LC-MS to resolve fungal burden, host response, and biomarker kinetics in pulmonary mucormycosis at complementary spatial and temporal scales. High-resolution MSI of *R. microsporus*–infected lungs identified Rhf and the previously unreported Hrhf as focal markers of hyphal proliferation, while simultaneously revealing rat neutrophil α-defensin “halos” and profound remodeling of anionic surfactant lipids in the surrounding host tissue.

These tissue-level biomarkers are directly coupled to non-invasive detection of siderophores in urine and proximal sampling of Rhf in human BAL fluid, thereby linking local metabolite production to systemic analytical signals. The detection of Rhf in BAL is consistent with the tissue MSI data showing focal Rhf production in infected lung lesions and supports the rationale for considering siderophore measurements alongside molecular tests in BAL or urine when mucormycosis is suspected.

The analytical advance lies in combining molecular specificity, spatial registration, and serial biofluid quantitation in a single workflow that can be adapted to other infection metallomics applications. Beyond defining a mechanistically coherent picture of the siderophore-neutrophil-surfactant axis in mucormycosis, our approach illustrates how infection metallomics and multimodal MSI can be harnessed to generate clinically relevant biomarkers and to dissect host-pathogen chemistry in situ. The same concept—mapping pathogen-derived metallophores alongside host effector molecules and lipids, then translating these signatures into body-fluid assays—should be broadly applicable to other siderophore-producing fungi, mycobacteria, and bacteria.

## Supplementary Information

Below is the link to the electronic supplementary material.

Supplementary file1 (PDF 4.35 MB)

## Data Availability

Raw mass spectrometry data can be downloaded from the permanent link https://hdl.handle.net/11104/0375910 and viewed using the open-source software CycloBranch.

## Declarations

### Animal welfare

All animal experiments were performed in accordance with the Czech Animal Protection Act (No. 246/1992) and were approved by the Czech Ministry of Education, Youth, and Sports (MSMT-9487/2019-5) and the Animal Welfare Committee of the Faculty of Medicine and Dentistry, Palacký University in Olomouc.

### Source of biological material

Rat lung and urine samples were obtained from the LEWIS rat pulmonary mucormycosis model described in this study. The human BAL specimen was obtained from a patient with pulmonary mucormycosis under the approval of the Ethical Panel of University Hospital Innsbruck.
