# Supplementary material for: Infection metallomics of pulmonary mucormycosis: multi-omics study of siderophore secretion, neutrophil recruitment, and lipid remodeling in rat lungs
Source: Anal Bioanal Chem. 2026 Jul 20;418(20):6763–75. doi: 10.1007/s00216-026-06658-0 (PMC13619744; doi:10.1007/s00216-026-06658-0)
Supplement: Supplementary file 1 [file 216_2026_6658_MOESM1_ESM.pdf]

Supplementary Information

Infection metallomics of pulmonary mucormycosis: multi-omics study of siderophore secretion, neutrophil recruitment, and lipid remodeling in rat lungs

Dominika Luptáková[1*], Tereza Hřivnová Juříková[1], Miloš Petřík[2,3,4], Kateřina Dvořáková Bendová[2], Barbora Neužilová[2], Helena Marešová[1], Andrea Palyzová[1], Oldřich Benada[1], Michaela Lackner[5], Petr Hubáček[6], Vladimír Havlíček[1*]

[1]*Institute of Microbiology of the Czech Academy of Sciences, Vídeňská 1083, 142 00 Prague, Czech Republic*

[2]*Institute of Molecular and Translational Medicine, Faculty of Medicine and Dentistry, Palacký University, Hněvotínská 5, 779 00 Olomouc, Czech Republic*

[3]*Czech Advanced Technology and Research Institute, Palacký University, Šlechtitelů 241/27, 779 00 Olomouc, Czech Republic*

[4]*Laboratory of Experimental Medicine, University Hospital, Hněvotínská 5, 779 00, Olomouc, Czech Republic*

[5]*Institute for Hygiene and Medical Microbiology, Medical University of Innsbruck, 6020 Innsbruck, Austria*

[6]*Department of the Medical Microbiology, 2nd Faculty of Medicine, Charles University and Motol University Hospital, V Úvalu 84, 150 06 Prague, Czech Republic*

* Corresponding authors:

Vladimír Havlíček, vlhavlic@biomed.cas.cz and Dominika Luptáková, dominika.luptakova@biomed.cas.cz

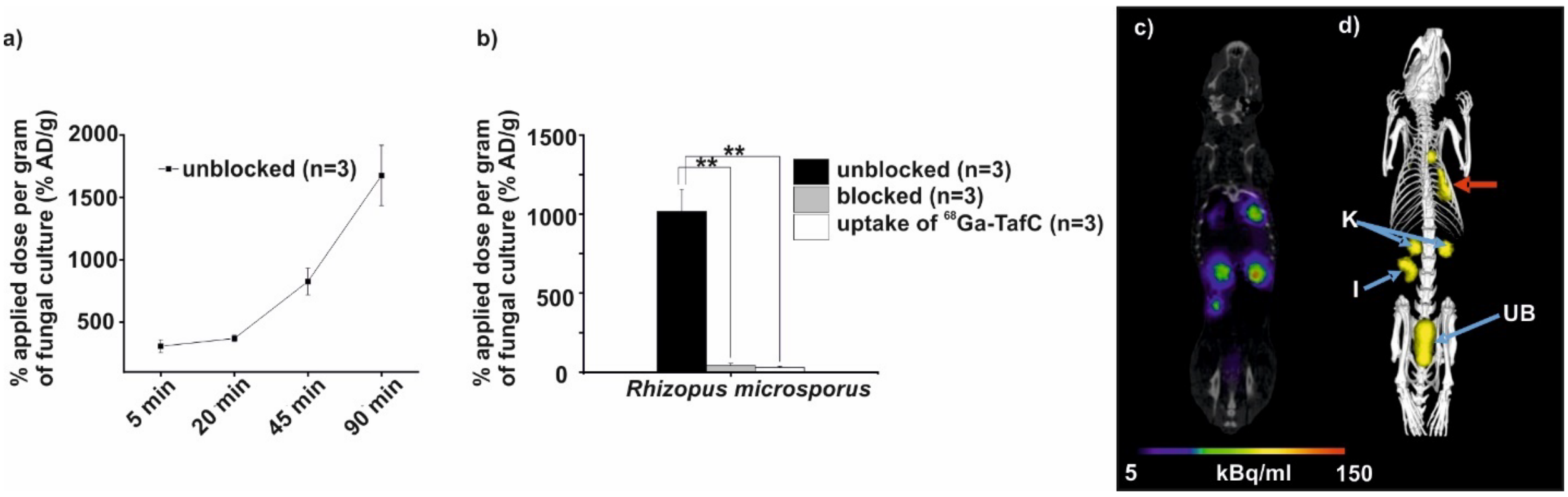

Fig. S1 *In vitro* and *in vivo* monitoring of ^{68}Ga-DfoB uptake by *R. microsporus*. (a) Time-dependent *in vitro* uptake of 68Ga-DfoB in *R. microsporus* fungal culture. (b) *In vitro* uptake of ^{68}Ga-DfoB in *R. microsporus* fungal culture under unblocked conditions, after blocking (preincubation with Fe-DfoB (15 mM) for 20 min), and uptake of ^{68}Ga-TafC (siderophore non-recognized/non-taken up by *R. microsporus*), all measured after 45 min of incubation. Static PET/CT images of a rat infected with *R. microsporus*, acquired 45 min after ^{68}Ga-DfoB injection: coronal slice (c) and 3D volume-rendered image (d). The red arrow indicates the site of infection. K, kidney; I, intestine; UB, urinary bladder; DfoB, desferrioxamine B; TafC, triacetylfusarinine C.

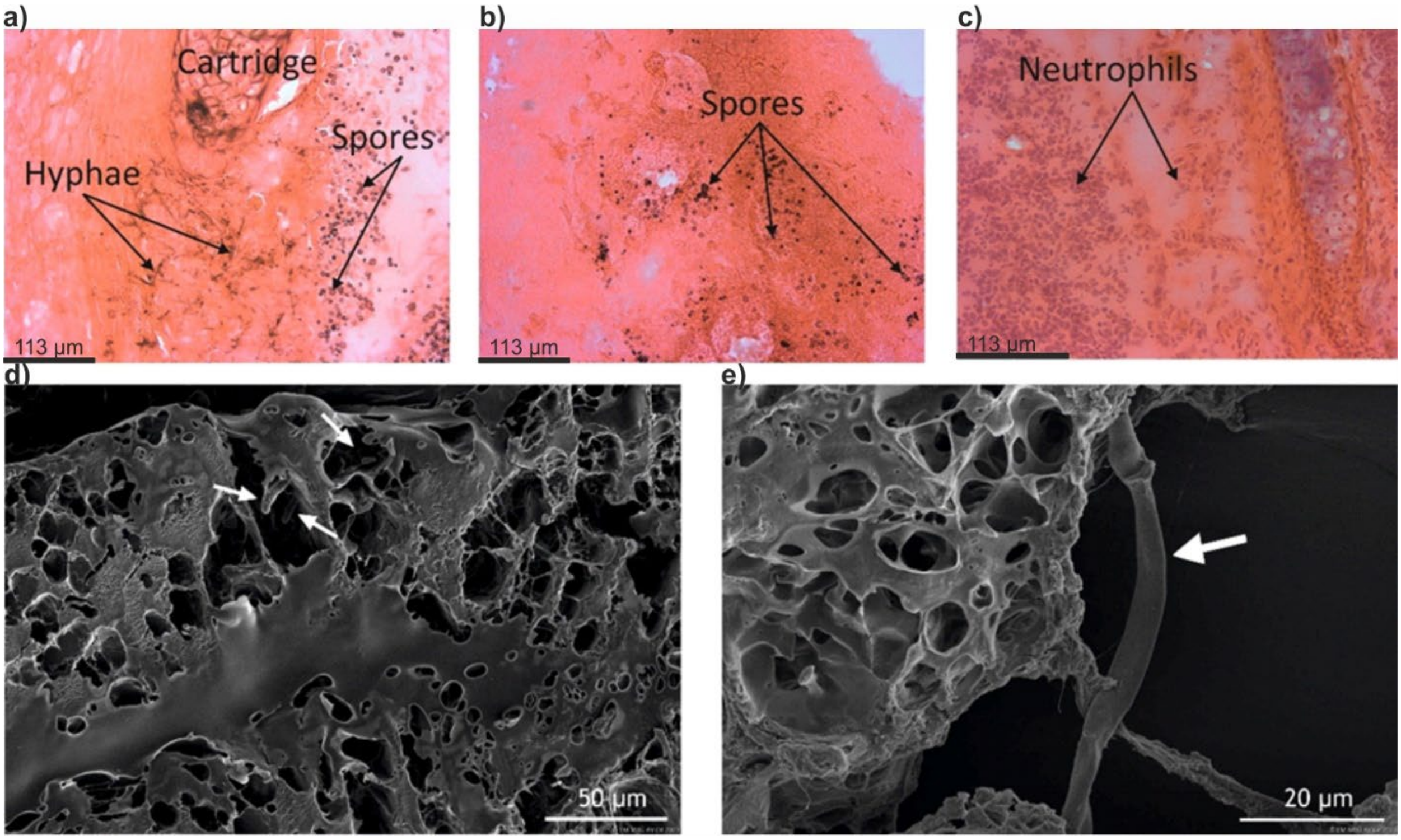

Fig. S2 Histological and scanning electron microscopic examination of *R. microsporus*–infected lung tissue. (a) Dense accumulation of Rhizopus spores and hyphae within the tracheal lumen and surrounding tracheal tissue visualized by optical microscopy after silver staining. (b) Fungal sporulation and proliferation in the lung parenchyma visualized by optical microscopy after silver staining. (c) Accumulation of neutrophils in the vicinity of tracheal area, which showed high density of fungal bodies, visualized by optical microscopy after hematoxylin and eosin staining. (d) Detailed imaging of lung tissue section infected with *R. microsporus* by scanning electron microscopy pointing to the presence of hyphae in the tissue (white arrow). (e) Zoom in the infected lung area displaying *R. microsporus* hypha (white arrow) perforating alveoli by scanning electron microscopy. Scanning electron microscopy observations were obtained from the same infection area, but from an adjacent consecutive tissue section to the histopathological examination.

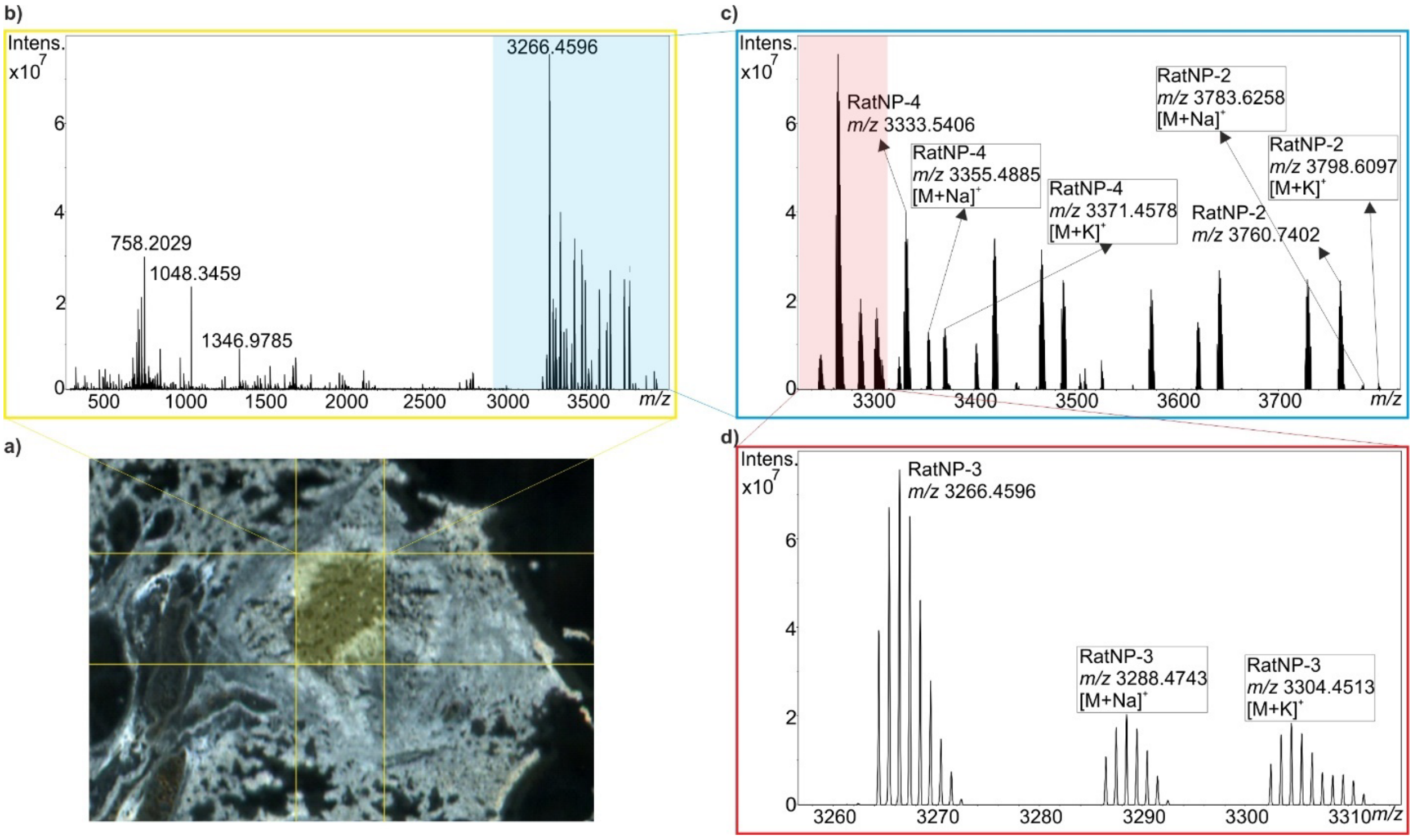

Fig. S3 Representative MALDI-MSI mass spectrum from an *R. microsporus*–infected lung region. (a) Optical image of the infected lung tissue with the analyzed area indicated in yellow, from which the mass spectrum was extracted. (b) Representative mass spectrum from *R. microsporus*–infected lung tissue acquired by MALDI-MSI in positive ion mode over an m/z range of 300–4000. (c) Zoomed-in view of the spectrum showing highly abundant peaks corresponding to annotated rat neutrophil antimicrobial peptides (RatNPs). (d) Further magnified view of the spectrum showing the isotopic envelopes of the RatNP-3 peptide in its protonated, sodium-adduct, and potassium-adduct forms.

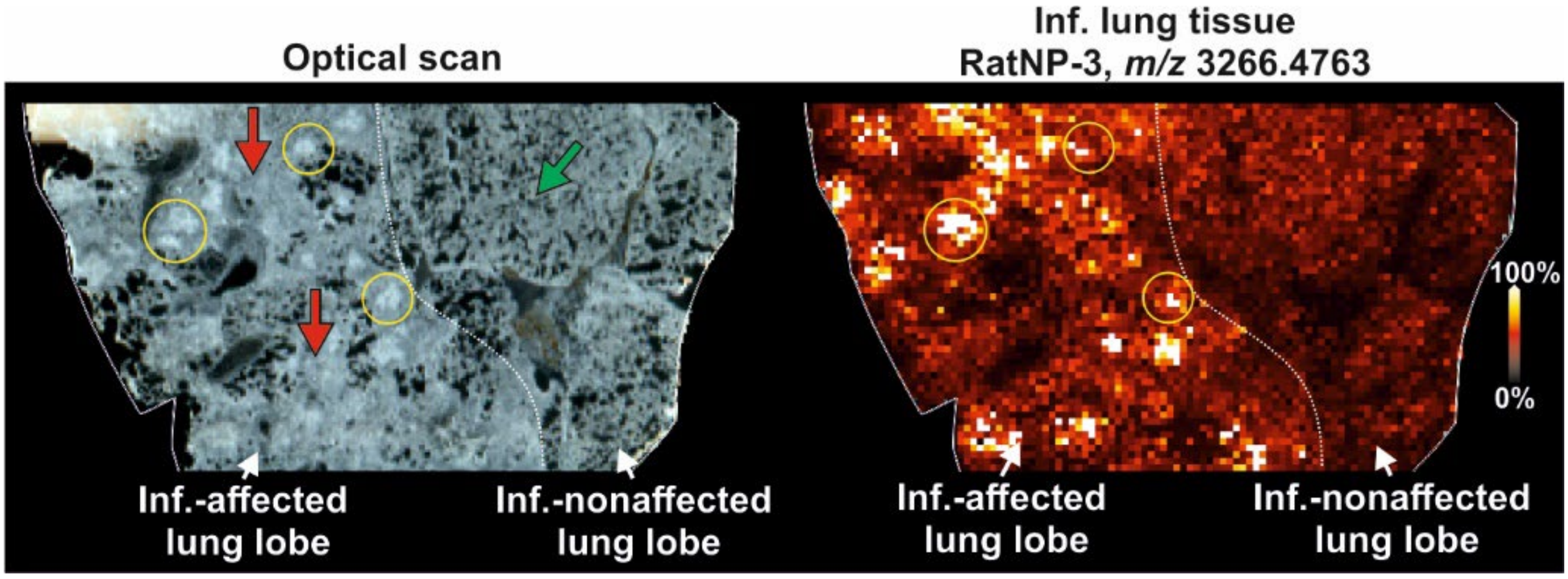

Fig. S4 Representative MALDI-MSI image showing the spatial distribution of the rat antimicrobial peptide RatNP-3 (m/z 3266.4763, $[M+H]^+$, M+2 isotope). In *R. microsporus*–affected regions, RatNP-3 is discretely localized and coincides with fungal foci (yellow circle), whereas in the infection-limited lung lobe its distribution is relatively uniform. Note that RatNP-2 and RatNP-4 exhibit the same distribution pattern as RatNP-3. The representative image was acquired with a raster step of 120 µm and normalized to the peak area of the molecular ion of mouse Hepcidin 25. Relative ion intensity is displayed using a fire color scale on a linear scale from 0 to 100%. In the infection-affected lung lobe, the tissue appeared condensed and formed a relatively uniform mass (red arrows), whereas the infection-nonaffected lung lobe retained a porous, sponge-like appearance (green arrow). Inf, infection.

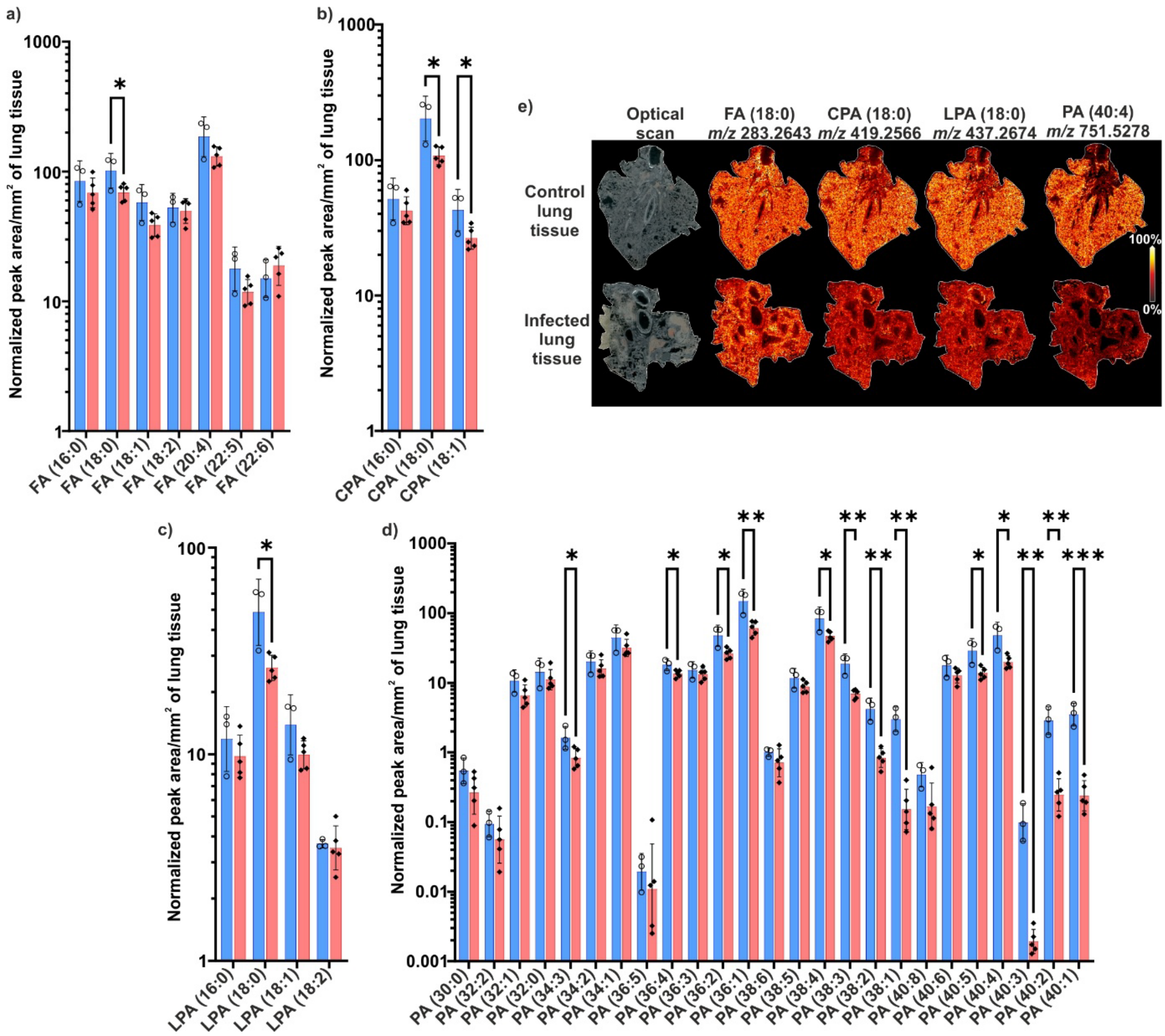

Fig. S5 *R. microsporus* infection–induced alterations in the lung lipid profile. Bar graphs show differences in the relative abundance of identified fatty acids (FA; a), cyclophosphatidic acids (CPA; b), lysophosphatidic acids (LPA; c), and phosphatidic acids (PA; d) between control (n = 3) and infected (n = 5) lung tissue groups. Data are expressed as mean ± standard deviation on a logarithmic scale. *, $p \leq 0.05$; **, $p \leq 0.01$; ***, $p \leq 0.001$; ****, $p \leq 0.0001$. (e) Ion images acquired by MALDI-MSI in negative ion mode showing the relative distribution of FA (18:0) (m/z 283.2643, $[M–H]^-$), CPA (18:0) (m/z 419.2566, $[M–H]^-$), LPA (18:0) (m/z 437.2674, $[M–H]^-$), and PA (40:4) (m/z 751.5278, $[M–H]^-$) in control and infected lung tissue. Representative images were acquired with a raster step of 120 µm and normalized using root-mean-square. Relative ion intensities are displayed using a fire color scale on a linear scale from 0 to 100%.

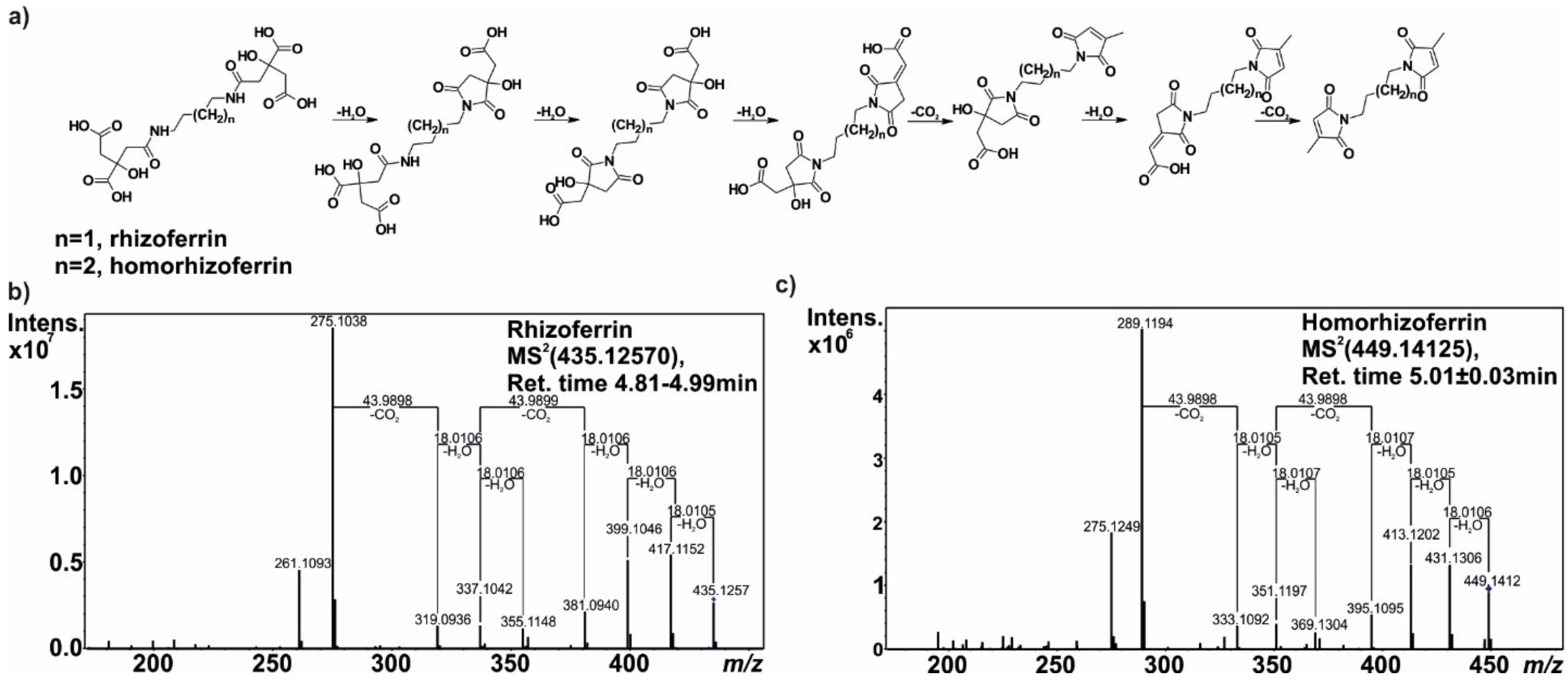

Fig. S6 Product ion spectra of rhizoferrin (Rhf) and homorhizoferrin (Hrhf) acquired by liquid chromatography–tandem mass spectrometry. (a) Proposed fragmentation pathway of deprotonated Rhf and Hrhf molecules, where n denotes the number of $–CH_2–$ groups. (b, c) Product ion scans of Rhf (b) and Hrhf (c) obtained by collision-induced dissociation at a collision energy of 10 V for both analytes. The fragmentation patterns are characterized by sequential neutral losses of H_2O and CO_2. Ret., retention; Intens., intensity.

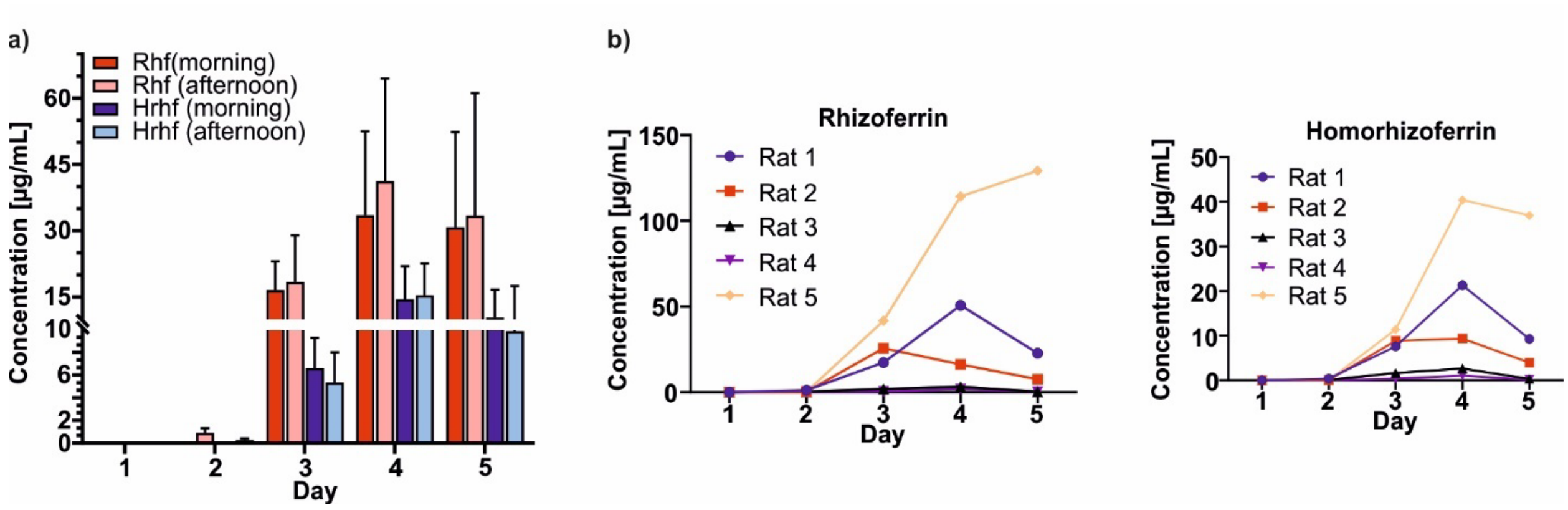

Fig. S7 Quantification of urinary rhizoferrin (Rhf) and homorhizoferrin (Hrhf) levels by liquid chromatography–mass spectrometry. (a) Quantification of Rhf and Hrhf in urine samples from *R. microsporus*–infected rats (n = 5), collected in the morning and afternoon over five consecutive days. Data are presented as mean ± standard deviation. (b) Concentrations of Rhf and Hrhf in urine from each rat (n = 5) over five days. Data represent the average of morning and afternoon urinary concentrations, each measured in three technical replicates. Rat 5 showed persistently high urinary siderophore concentrations through day 5; in the corresponding tissue analyses, this animal showed the most extensive pulmonary involvement and the weakest RatNP signal among the infected animals.

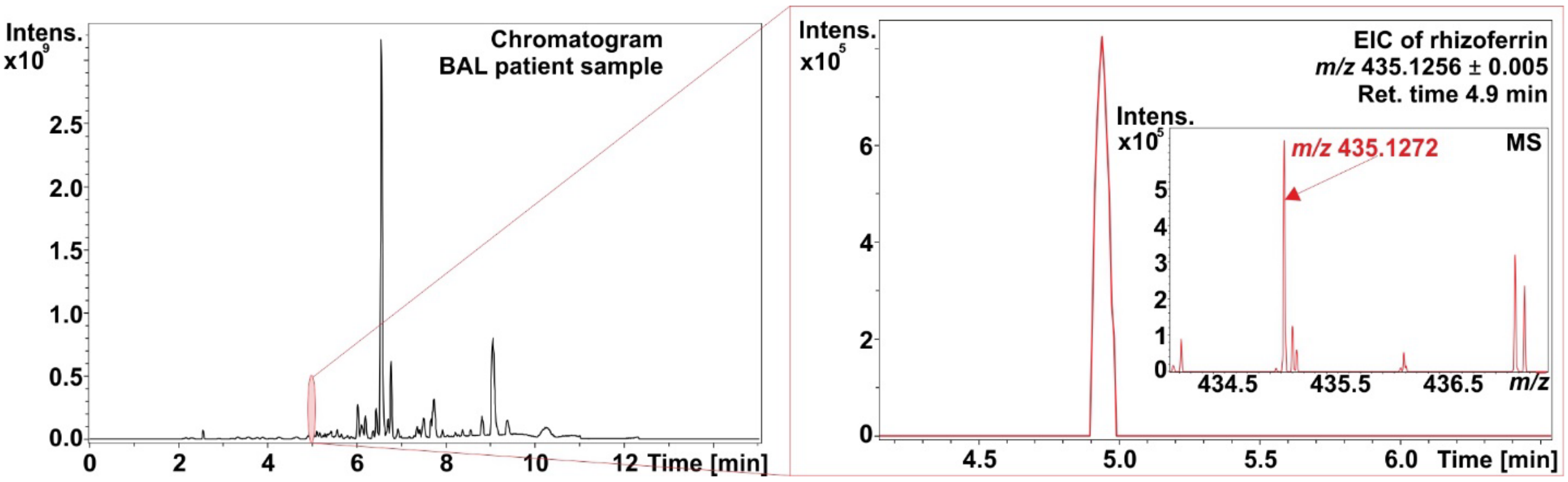

Fig. S8 Liquid chromatography-mass spectrometry analysis of a human bronchoalveolar lavage sample from a patient with pulmonary mucormycosis. Shown is the chromatogram from liquid chromatography-mass spectrometry analysis (left) of the bronchoalveolar lavage sample extract, together with a zoomed extracted ion chromatogram for m/z 435.1256 ± 0.005 and an inset displaying the corresponding enlarged mass spectrum. BAL, bronchoalveolar lavage; EIC, extracted ion chromatogram; Intens., intensity; Ret., retention; MS, mass spectrum.

Table S1 Validation parameters of the liquid chromatography–mass spectrometry method used for quantifying total rhizoferrin. The analytical performance characteristics evaluated included linearity, calibration range, coefficient of determination (R^2), method limit of detection (mLOD), and method limit of quantification (mLOQ). These parameters were established using calibration standards at seven different concentrations and blanks measured in six technical replicates. Data normalization prior to curve fitting was omitted due to the unstable signal intensity of the internal standard ferricrocin (data not shown) and the robust linear response of desferri-rhizoferrin over the tested concentration range.

	**Rhizoferrin**
**Linear regression equation**	y = 243657x
**Linear calibration range [ng/mL]**	5 - 1000
**Coefficient of determination (R^2)**	0.9985
**mLOD [µg/mL]**	0.008
**mLOQ [µg/mL]**	0.026

Table S2 List of assigned rat antimicrobial peptides (RatNPs) with high mass accuracy as determined by MALDI-FTICR-MSI experiments.

Rat defensins	Peptide sequence	Disulfide bonds	Molecular formula	Observed *m/z*, $[M+H]^+$, M+2 isotope	Theoretical *m/z*, $[M+H]^+$, M+2 isotope	Error ppm
**RatNP-3**	CSCRTSSCRFGERLSGACRLNGRIYRLCC	3	$C_{130}H_{215}N_{48}O_{39}S_6$	3266.4596	3266.4672	-2.3
**RatNP-4**	ACYCRIGACVSGERLTGACGLNGRIYRLCCR	3	$C_{136}H_{224}N_{47}O_{39}S_6$	3333.5406	3333.5347	1.7
**RatNP-2**	VTCYCRSTRCGFRERLSGACGYRGRIYRLCCR	3	$C_{155}H_{249}N_{56}O_{42}S_6$	3760.7402	3760.7430	-0.8

Table S3 Comparison of relative intensities of rat antimicrobial peptides between control (ctrl, n = 3) and infected (inf, n = 5) lung tissue samples. Data were analyzed using an ordinary one-way ANOVA followed by uncorrected Fisher's LSD multiple comparison test, with a single pooled variance. Statistical significance is reported as exact p-values, with results considered significant at p ≤ 0.05 (values in bold).

**Multiple comparison ctrl vs. inf p-value**	**Ctrl RatNP-2**	**Ctrl RatNP-3**	**Ctrl RatNP-4**	**Inf RatNP-2**	**Inf RatNP-3**	**Inf RatNP-4**
**Ctrl RatNP-2**		0.4629	0.8570	**0.0114**	n.a.	n.a.
**Ctrl RatNP-3**	0.4629		0.5775	n.a.	**<0.0001**	n.a.
**Ctrl RatNP-4**	0.8570	0.5775		n.a.	n.a.	**0.0002**
**Inf RatNP-2**	**0.0114**	n.a.	n.a.		**<0.0001**	**0.0310**
**Inf RatNP-3**	n.a.	**<0.0001**	n.a.	**<0.0001**		**<0.0001**
**Inf RatNP-4**	n.a.	n.a.	**0.0002**	**0.0310**	**<0.0001**	

Table S4 Pearson correlation analysis of the *R. microsporus* siderophores rhizoferrin and homorhizoferrin with immune system–derived rat antimicrobial peptides (RatNPs) and heme b in infected lung tissues (n = 5). Correlations were calculated from data obtained from the same or consecutive tissue sections. Results were considered significant at $p \leq 0.05$ (values in red). The strength and direction of the linear relationship between variables are expressed as r-values (values in bold italics).

**p-value<br>Pearson r<br>[Confidence interval of r]**	**Rhizoferrin**	**Homorhizoferrin**	**RatNP-3**	**RatNP-4**	**RatNP-2**	**Heme b**
**Rhizoferrin**		**0.002**<br>***0.986***<br>**[0.801-0.999]**	0.204<br>*-0.682*<br>[-0.977-0.503]	0.100<br>*-0.806*<br>[-0.987-0.265]	0.138<br>*-0.758*<br>[-0.9829-0.3751]	**0.046**<br>***0.885***<br>**[0.0145-0.992]**
**Homorhizoferrin**	**0.002**<br>***0.986***<br>**[0.801-0.999]**		0.242<br>*-0.643*<br>[-0.973-0.553]	0.169<br>*-0.722*<br>[-0.980-0.442]	0.201<br>*-0.686*<br>[-0.9769-0.4977]	0.095<br>*0.812*<br>[-0.247-0.987]
**RatNP-3**	0.204<br>*-0.682*<br>[-0.977-0.503]	0.242<br>*-0.643*<br>[-0.973-0.553]		**0.039**<br>***0.896***<br>**[0.067-0.993]**	**0.010**<br>***0.960***<br>**[0.5063-0.9974]**	0.418<br>*-0.475*<br>[-0.957-0.701]
**RatNP-4**	0.100<br>*-0.806*<br>[-0.987-0.265]	0.169<br>*-0.722*<br>[-0.980-0.442]	**0.039**<br>***0.896***<br>**[0.067-0.993]**		**0.007**<br>***0.969***<br>**[0.5957-0.9980]**	0.120<br>*-0.780*<br>[-0.985-0.328]
**RatNP-2**	0.138<br>*-0.758*<br>[-0.983-0.375]	0.201<br>*-0.686*<br>[-0.977-0.498]	**0.010**<br>***0.960***<br>**[0.506-0.997]**	**0.007**<br>***0.969***<br>**[0.596-0.998]**		0.225<br>*-0.660*<br>[-0.975-0.532]
**Heme b**	**0.038**<br>***0.898***<br>**[0.076-0.993]**	0.087<br>*0.823*<br>[-0.216-0.988]	0.358<br>*-0.531*<br>[-0.962-0.661]	0.090<br>*-0.819*<br>[-0.988-0.227]	0.182<br>*-0.707*<br>[-0.979-0.466]	

Table S5 List of assigned fatty acids and glycerophospholipid species with high mass accuracy (mass error < 2 ppm) obtained from MALDI-FTICR-MSI experiments. Results of multiple comparisons of the relative intensities of annotated fatty acids and lipid species between control (ctrl, n = 3) and infected (inf, n = 5) lung tissue samples, performed using an unpaired t test, are reported as exact p-values. Results were considered significant at p ≤ 0.05 (values in bold).

Species identification	Molecular Formula	Measured *m/z*, [M-H]-	Theoretical *m/z*, [M-H]-	Error ppm	Multiple comparison ctrl vs. inf p-value
FA (16:0)	$C_{16}H_{32}O_2$	255.23298	255.232954	0.10	0.334965
FA (18:0)	$C_{18}H_{36}O_2$	283.26425	283.264254	-0.01	**0.043796**
FA (18:1)	$C_{18}H_{34}O_2$	281.24852	281.248604	-0.30	0.055649
FA (18:2)	$C_{18}H_{32}O_2$	279.23291	279.232954	-0.16	0.721327
FA (20:4)	$C_{20}H_{32}O_2$	303.23292	303.232954	-0.1	0.078269
FA (22:5)	$C_{22}H_{34}O_2$	329.24851	329.248604	-0.29	0.078757
FA (22:6)	$C_{22}H_{32}O_2$	327.23282	327.232954	-0.41	0.358093
CPA (16:0)	$C_{19}H_{37}O_6P$	391.22546	391.225499	0.10	0.322403
CPA (18:0)	$C_{21}H_{41}O_6P$	419.25655	419.256800	0.59	**0.016613**
CPA (18:1)	$C_{21}H_{39}O_6P$	417.24096	417.241150	0.46	**0.037826**
LPA (16:0)	$C_{19}H_{39}O_7P$	409.23642	409.236064	0.87	0.332520
LPA (18:0)	$C_{21}H_{43}O_7P$	437.26737	437.267364	0.01	**0.015636**
LPA (18:1)	$C_{21}H_{41}O_7P$	435.25208	435.251714	0.84	0.075917
LPA (18:2)	$C_{21}H_{39}O_7P$	433.23578	433.236064	-0.65	0.878584
PA (30:0)	$C_{33}H_{65}O_8P$	619.43474	619.434430	0.50	0.117338
PA (32:0)	$C_{35}H_{69}O_8P$	647.46545	647.465730	-0.43	0.378931
PA (32:1)	$C_{35}H_{67}O_8P$	645.44996	645.450080	-0.19	0.378931
PA (32:2)	$C_{35}H_{65}O_8P$	643.43446	643.434430	0.05	0.453544
PA (34:0)	$C_{37}H_{73}O_8P$	675.49654	675.497030	-0.73	**0.006801**
PA (34:1)	$C_{37}H_{71}O_8P$	673.48090	673.481380	-0.71	0.194923
PA (34:2)	$C_{37}H_{69}O_8P$	671.46534	671.465730	-0.58	0.343383
PA (34:3)	$C_{37}H_{67}O_8P$	669.45007	669.450080	-0.01	**0.041733**
PA (36:1)	$C_{39}H_{75}O_8P$	701.51223	701.512680	-0.64	**0.008302**
PA (36:2)	$C_{39}H_{73}O_8P$	699.49653	699.497030	-0.71	**0.017571**
PA (36:3)	$C_{39}H_{71}O_8P$	697.48102	697.481380	-0.52	0.403037
PA (36:4)	$C_{39}H_{69}O_8P$	695.46555	695.465730	-0.26	**0.032294**
PA (36:5)	$C_{39}H_{67}O_8P$	693.44977	693.450080	-0.45	0.823024
PA (38:1)	$C_{41}H_{79}O_8P$	729.54400	729.543980	0.03	**0.001131**
PA (38:2)	$C_{41}H_{77}O_8P$	727.52816	727.528330	-0.23	**0.001346**
PA (38:3)	$C_{41}H_{75}O_8P$	725.51270	725.512680	0.03	**0.002675**
PA (38:4)	$C_{41}H_{73}O_8P$	723.49662	723.497030	-0.57	**0.022320**
PA (38:5)	$C_{41}H_{71}O_8P$	721.48092	721.481380	-0.64	0.143024
PA (38:6)	$C_{41}H_{69}O_8P$	719.46546	719.465730	-0.38	0.293217
PA (40:1)	$C_{43}H_{83}O_8P$	757.57513	757.575280	-0.20	**0.000924**
PA (40:2)	$C_{43}H_{81}O_8P$	755.55960	755.559630	-0.04	**0.002642**
PA (40:3)	$C_{43}H_{79}O_8P$	753.54400	753.543980	0.03	**0.008977**
PA (40:4)	$C_{43}H_{77}O_8P$	751.52778	751.528330	-0.73	**0.010605**
PA (40:5)	$C_{43}H_{75}O_8P$	749.51222	749.512680	-0.61	**0.013732**
PA (40:6)	$C_{43}H_{73}O_8P$	747.49652	747.497030	-0.68	0.130237
PA (40:8)	$C_{43}H_{69}O_8P$	743.46555	743.465730	-0.24	0.111590
PG (30:0)	$C_{36}H_{71}O_{10}P$	693.47086	693.471209	-0.50	**0.006865**
PG (32:0)	$C_{38}H_{75}O_{10}P$	721.50185	721.502509	-0.91	0.052564
PG (32:1)	$C_{38}H_{73}O_{10}P$	719.48640	719.486859	-0.64	**0.017588**
PG (34:1)	$C_{40}H_{77}O_{10}P$	747.51761	747.518159	-0.73	0.072977
PG (34:2)	$C_{40}H_{75}O_{10}P$	745.50204	745.502509	-0.63	0.089718

PG (34:3)	$C_{40}H_{73}O_{10}P$	743.48661	743.486859	-0.33	0.072760
PG (36:1)	$C_{42}H_{81}O_{10}P$	775.54946	775.549459	0.00	0.230662
PG (36:2)	$C_{42}H_{79}O_{10}P$	773.53336	773.533809	-0.58	0.337253
PG (36:3)	$C_{42}H_{77}O_{10}P$	771.51777	771.518159	-0.50	0.404450
PG (36:4)	$C_{42}H_{75}O_{10}P$	769.50181	769.502509	-0.91	0.273645
PG (36:5)	$C_{42}H_{73}O_{10}P$	767.48642	767.486859	-0.57	0.287019
PG (38:4)	$C_{44}H_{79}O_{10}P$	797.53344	797.533809	-0.46	0.374585
PG (38:5)	$C_{44}H_{77}O_{10}P$	795.51762	795.518159	-0.68	0.283161
PG (38:6)	$C_{44}H_{75}O_{10}P$	793.50211	793.502509	-0.50	0.928061
PG (40:6)	$C_{46}H_{79}O_{10}P$	821.53331	821.533809	-0.61	0.192772
PG (40:7)	$C_{46}H_{77}O_{10}P$	819.51776	819.518159	-0.49	0.074460
PG (40:8)	$C_{46}H_{75}O_{10}P$	817.50169	817.502509	-1.00	**0.028080**
PG (42:10)	$C_{48}H_{75}O_{10}P$	841.50161	841.502509	-1.07	**0.004641**
PG (44:12)	$C_{50}H_{75}O_{10}P$	865.50157	865.502509	-1.08	**0.014662**
PI (32:0)	$C_{41}H_{79}O_{13}P$	809.51902	809.518553	0.58	0.504130
PI (32:1)	$C_{41}H_{77}O_{13}P$	807.50258	807.502903	-0.40	0.084626
PI (32:2)	$C_{41}H_{75}O_{13}P$	805.48730	805.487253	0.06	0.211684
PI (34:1)	$C_{43}H_{81}O_{13}P$	835.53359	835.534203	-0.73	0.172581
PI (34:2)	$C_{43}H_{79}O_{13}P$	833.51819	833.518553	-0.43	**0.014891**
PI (34:3)	$C_{43}H_{77}O_{13}P$	831.50215	831.502903	-0.91	**0.011618**
PI (34:4)	$C_{43}H_{75}O_{13}P$	829.48670	829.487253	-0.67	0.189994
PI (36:1)	$C_{45}H_{85}O_{13}P$	863.56483	863.565503	-0.78	0.100231
PI (36:2)	$C_{45}H_{83}O_{13}P$	861.54893	861.549853	-1.07	**0.015538**
PI (36:3)	$C_{45}H_{81}O_{13}P$	859.53363	859.534203	-0.67	0.127165
PI (36:4)	$C_{45}H_{79}O_{13}P$	857.51800	857.518553	-0.64	0.340456
PI (36:5)	$C_{45}H_{77}O_{13}P$	855.50272	855.502903	-0.21	**0.039648**
PI (38:3)	$C_{47}H_{85}O_{13}P$	887.56550	887.565503	0.00	0.844502
PI (38:4)	$C_{47}H_{83}O_{13}P$	885.54867	885.549853	-1.33	0.592674
PI (38:5)	$C_{47}H_{81}O_{13}P$	883.53324	883.534203	-1.09	0.717435
PI (38:6)	$C_{47}H_{79}O_{13}P$	881.51785	881.518553	-0.80	0.167143
PI (40:4)	$C_{49}H_{87}O_{13}P$	913.58103	913.581153	-0.13	0.113670
PI (40:5)	$C_{49}H_{85}O_{13}P$	911.56497	911.565503	-0.58	0.225961
PI (40:6)	$C_{49}H_{83}O_{13}P$	909.54908	909.549853	-0.85	**0.033654**
PI (40:7)	$C_{49}H_{81}O_{13}P$	907.53393	907.534203	-0.30	**0.002337**
